# *Valsa mali* secretes an effector protein VmEP1 to target a K homology domain‐containing protein for virulence in apple

**DOI:** 10.1111/mpp.13248

**Published:** 2022-07-18

**Authors:** Weidong Wang, Shuaile Wang, Wan Gong, Luqiong Lv, Liangsheng Xu, Jiajun Nie, Lili Huang

**Affiliations:** ^1^ State Key Laboratory of Crop Stress Biology for Arid Areas Yangling China; ^2^ College of Plant Protection Northwest A&F University Yangling China

**Keywords:** effector protein, plant immunity, protein stability, RNA‐binding protein, *Valsa mali*

## Abstract

The K homology (KH) repeat is an RNA‐binding motif that exists in various proteins, some of which participate in plant growth. However, the function of KH domain‐containing proteins in plant defence is still unclear. In this study, we found that a KH domain‐containing protein in apple (*Malus domestica*), HEN4‐like (MdKRBP4), is involved in the plant immune response. Silencing of *MdKRBP4* compromised reactive oxygen species (ROS) production and enhanced the susceptibility of apple to *Valsa mali*, whereas transient overexpression of *MdKRBP4* stimulated ROS accumulation in apple leaves, indicating that MdKRBP4 is a positive immune regulator. Additionally, MdKRBP4 was proven to interact with the VmEP1 effector secreted by *V. mali*, which led to decreased accumulation of MdKRBP4. Coexpression of MdKRBP4 with VmEP1 inhibited cell death and ROS production induced by MdKRBP4 in *Nicotiana benthamiana*. These results indicate that MdKRBP4 functions as a novel positive regulatory factor in plant immunity in *M. domestica* and is a virulence target of the *V. mali* effector VmEP1.

## Graphical abstract

This study demonstrated that the effector protein VmEP1 secreted by *Valsa mali* interferes with the apple plant immune response by promoting the degradation of MdKRBP4.

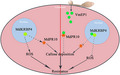

## 1 INTRODUCTION

In the natural environment plants encounter various microorganisms, some of which are pathogenic. Due to a lack of specific immune cells, plants have developed a complex immune mechanism composed of microbe‐associated molecular pattern (MAMP)/pathogen‐associated molecular pattern (PAMP)‐triggered immunity (PTI) and effector‐triggered immunity (ETI) to resist pathogens (Jones & Dangl, 2006; Macho & Zipfel, 2014). The activation of plant immunity leads to a series of reactions, including an increase in reactive oxygen species (ROS), Ca^2+^ influx, mitogen‐activated protein kinase activation, and regional cell death (Boller & Felix, 2009; Cheng et al., 2019; Spoel & Dong, 2012; Zipfel & Oldroyd, 2017). Numerous proteins, such as the transcription factor BZR1, which is involved in brassinosteroid signalling (Qi et al., 2021), mitogen‐activated protein kinase kinases (Rufian et al., 2021), and FLOWERING LOCUS KH domain (FLK) (Fabian et al., 2021), have been reported to regulate plant immune responses. Among these, FLK is an RNA‐binding protein (RBP) that regulates plant development (Lim et al., 2004), the ROS‐scavenging enzyme catalase, and plant immunity (Fabian et al., 2021). Therefore, we aimed to investigate the role of RBPs in pathogen defence and ROS regulation.

RBPs are pivotal factors manipulating posttranscriptional RNA metabolism during plant growth and stress responses (Lee & Kang, 2016). The K homology (KH) domain‐containing proteins are ancient RBPs found in diverse organisms (Nicastro et al., 2015). A few KH domain‐containing proteins are involved in RNA metabolism, such as pre‐mRNA processing, mRNA stabilization (Rodriguez‐Cazorla et al., 2015), and microRNA biogenesis (Karlsson et al., 2015). KH domain‐containing proteins, such as SPL11‐interacting protein1 (SPIN1) (Vega‐Sanchez et al., 2008), HUA ENHANCER4 (HEN4) (Cheng et al., 2003; Ortuno‐Miquel et al., 2019), FLK (Lim et al., 2004), PEPPER (PEP) (Ripoll et al., 2006), KHZ1, and KHZ2 (Yan et al., 2017), have been shown to control flowering. Furthermore, some KH domain‐containing proteins are crucial for the stress response, such as resistance against *Fusarium oxysporum* (Thatcher et al., 2015), tomato mosaic virus (Fujisaki & Ishikawa, 2008), and abiotic stress (Chen et al., 2013; Guan et al., 2013). However, the role of KH domain‐containing proteins in manipulating development in plants has only been reported *Arabidopsis thaliana*. Moreover, only a few studies have correlated KH domain‐containing proteins with the plant immune response. Only *A. thaliana* FLK is known to regulate resistance against pathogenic bacteria (Fabian et al., 2021; Lim et al., 2004). Whether other KH domain‐containing proteins are involved in regulating host resistance is still unclear. Therefore, it is necessary to identify other KH domain‐containing proteins and examine their possible involvement in the plant immune response.

Typically, to counteract the plant immune system and establish a successful infection during colonization, pathogenic microorganisms deploy effectors to avoid host recognition and inhibit the host defence response (Pradhan et al., 2021). These effector molecules manipulate the immune response by targeting key host proteins, such as lectin‐like receptor kinase protein (Kanzaki et al., 2008), E3 ubiquitin ligase (Bos et al., 2010; Park et al., 2012), BAK1‐associated receptor complexes (Lu et al., 2010), protein kinase (Murphy et al., 2018; Tanaka et al., 2014), exocyst component Sec5 (Du et al., 2015), peroxidase (Hemetsberger et al., 2012; Zhang et al., 2015), protein phosphatase 1 (Boevink et al., 2016), endoplasmic reticulum (ER)‐luminal binding immunoglobulin proteins (Jing et al., 2016), cytoplasmic transacetylase (Li et al., 2018), and WRKY transcription factors (Ma et al., 2021), which regulate diverse aspects of plant cell development and metabolism. However, pathogen effectors rarely target KH domain‐containing proteins.

The ascomycete *Valsa mali*, which causes apple (*Malus domestica*) Valsa canker, secretes effector protein 1 (VmEP1) to inhibit BAX‐induced programmed cell death (Li et al., 2015) and targets host pathogenesis‐related protein 10 (PR10) (Wang et al., 2021). However, the mechanism by which VmEP1 manipulates plant immunity is not known. Previously, we found that VmEP1 targeted MdPR10 (Wang et al., 2021) and other host proteins, such as KH domain‐containing protein HEN4‐like (MdKRBP4). In the present study, we discovered that VmEP1 interacts with MdKRBP4 and promotes the degradation of MdKRBP4. Overexpression of *MdKRBP4* induced ROS production and increased the transcription level of genes encoding pathogenesis‐related proteins, such as *MdPR1*, *MdPR2*, *MdPR5*, and *MdPR10*. Silencing of *MdKRBP4* in apples enhanced the susceptibility to *V. mali*, indicating the role of MdKRBP4 in positively regulating plant immunity. Furthermore, coexpression of *VmEP1* and *MdKRBP4* inhibited MdKRBP4‐induced cell death in *Nicotiana benthamiana*. Our data collectively indicate that VmEP1 suppresses plant immunity by promoting MdKRBP4 degradation.

## 2 RESULTS

### 2.1 MdKRBP4 interacts with VmEP1

**FIGURE 1:**
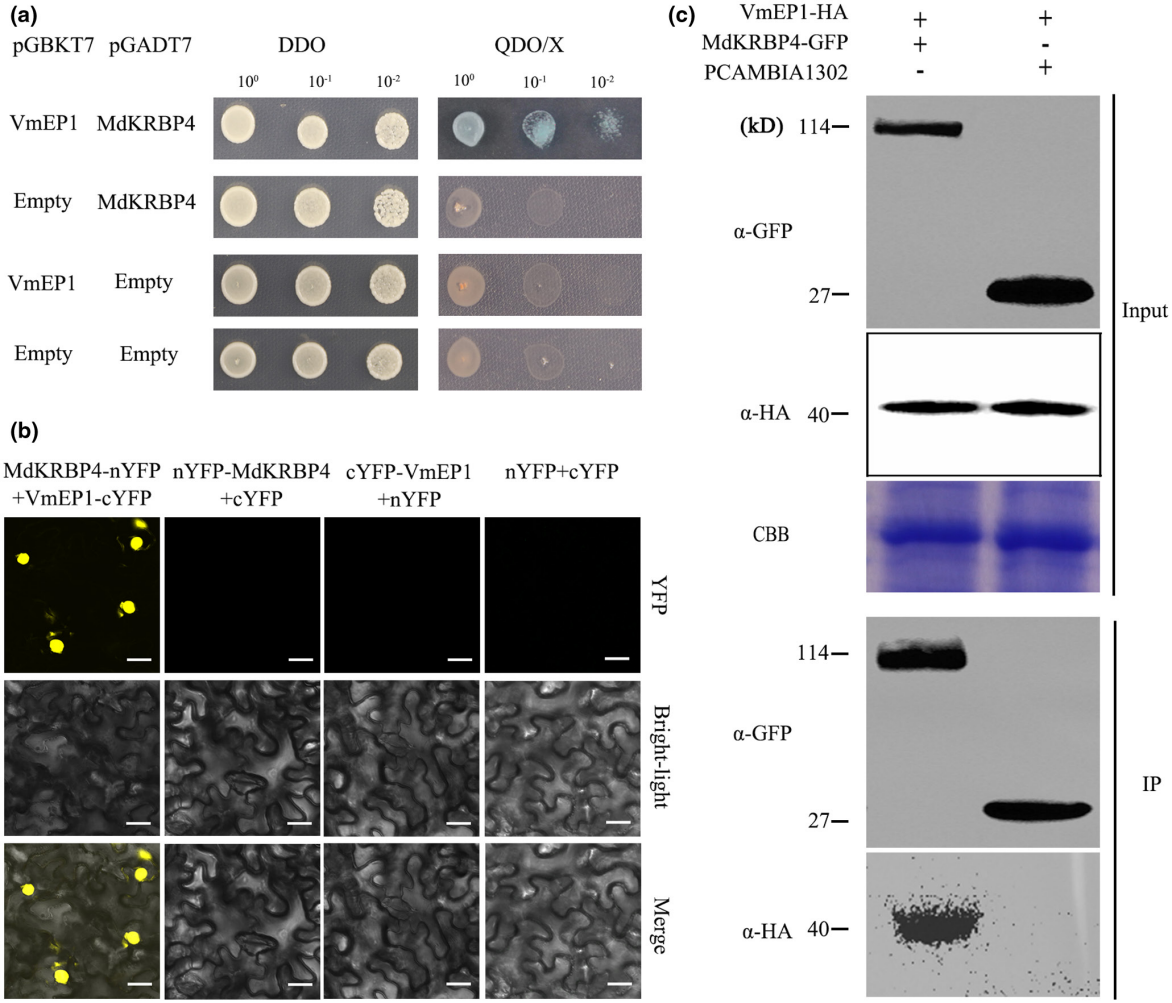
VmEP1 interacts with MdKRBP4 in vivo. (a) The interaction of VmEP1 with MdKRBP4 in a GAL4 yeast two‐hybrid system. VmEP1 interacts with MdKRBP4, as indicated by yeast two‐hybrid assays. Double dropout (DDO) medium is SD −Leu/−Trp medium. Quadruple dropout (QDO) medium/X is SD −Ade/−His/−Leu/−Trp/+X‐α‐Gal medium. (b) Bimolecular fluorescence complementation assays showing the interaction of VmEP1 with MdKRBP4 in *Nicotiana benthamiana* leaves. *Agrobacterium tumefaciens* containing *VmEP1*‐cYFP/nYFP*‐MdKRBP4* were coinfiltrated in *N. benthamiana* leaves. *VmEP1*‐cYFP/nYFP, nYFP‐*MdKRBP4*/cYFP, and nYFP/cYFP were used as negative controls. Bar = 20 μm. (c) Coimmunoprecipitation assays using anti‐GFP magnetic beads. Pairs of *VmEP1*‐*HA* and *MKRBP4*‐*GFP*/*GFP* were coexpressed in *N. benthamiana* leaves. The immunoprecipitated proteins were resolved by SDS‐PAGE and analysed by western blot with the anti‐GFP or anti‐HA antibody. Molecular mass markers are shown (in kilodaltons). Blots were stained with Coomassie brilliant blue (CBB) to verify equal loading. This assay was repeated three times.

In our previous study, the secretory protein VmEP1, with no known function, was shown to suppress plant defence by interrupting MdPR10‐mediated resistance against *V. mali* (Wang et al., 2021). In the present study, *VmEP1‐GFP* was expressed in *N. benthamiana* leaves and the fusion proteins and associated proteins were purified with anti‐GFP magnetic beads. To uncover the virulence mechanism of VmEP1 in *V. mali*, liquid chromatography–tandem mass spectrometry analysis of the purified VmEP1 and related proteins detected peptides of KH domain‐containing proteins (Table 1), with no peptides after purification of green fluorescent protein (GFP) alone. The yeast two‐hybrid (Y2H) assay showed that VmEP1 targets a KH domain‐containing protein (Figure 1a, Table S1). Therefore, the KH domain‐containing protein, named MdKRBP4, was identified as one of the best candidates.

**TABLE 1:** Candidate proteins associated with VmEP1 in plants

Accession	Description
A0A1S3XHV7	ABC transporter B family member 25‐like
A0A1U7VME9	Mitogen‐activated protein kinase 12‐like
A0A1U7YIC1	LRR receptor‐like serine/threonine protein kinase
A0A1S4A6X4	Serine/threonine protein kinase WNK8‐like isoform X1
A0A1U7Y2B6	Cysteine‐rich receptor‐like protein kinase 42
A0A1S4AJR8	eIF‐2α kinase activator GCN1 isoform X2
A0A1U7X8D3	NAD kinase 2, chloroplastic‐like isoform X2
A0A1J6KK09	E3 ubiquitin protein ligase
A0A1S3Z289	RING‐type E3 ubiquitin transferase
A0A1U7WCS0	Putative E3 ubiquitin protein ligase
A0A1S4BVK9	E3 ubiquitin protein ligase RNF170‐like
A0A1U7XI91	RING‐type E3 ubiquitin transferase
A0A1S3X1B4	E3 ubiquitin protein ligase RLIM‐like
A0A1U7V537	ABC transporter G family member 14‐like
A0A1U7V720	KH domain‐containing protein
A0A1U7W6H0	Zinc finger MYM‐type protein 1‐like
A0A1J6L7B6	Ethylene overproduction protein 1

Furthermore, a bimolecular fluorescence complementation (BiFC) assay was performed to verify the association between MdKRBP4 and VmEP1 from different species. The *MdKRBP4‐nYFP* and *VmEP1‐cYFP* constructs were transformed into *N. benthamiana* leaves using *Agrobacterium tumefaciens*. The empty vectors (nYFP or cYFP) were used as the negative controls. In contrast to the control, the cells coexpressing *MdKRBP4‐nYFP* and *VmEP1‐cYFP* displayed a robust yellow fluorescence signal in the nucleus, suggesting an interaction between VmEP1 and MdKRBP4. Meanwhile, no fluorescence was detected in the negative control (Figure 1b). In addition, we tested the interaction between cYFP and nYFP empty vectors; yellow fluorescence was not detected (Figure 1d). Further analysis performed to validate the results of the BiFC assay revealed VmEP1 localization in the nucleus, cytoplasm, and plasma membrane (Figure S1a), and MdKRBP4 localization in the nucleus (Figure S1b). These results suggest that VmEP1 interacts with MdKRBP4 in the nucleus.

Subsequently, VmEP1‐HA was coexpressed with MdKRBP4‐GFP in *N. benthamiana* leaves, using GFP as a control, and a coimmunoprecipitation (Co‐IP) assay was performed using anti‐GFP magnetic beads. Proteins in each sample were detected by western blot with anti‐GFP and anti‐HA antibodies. The results indicate that all genes were successfully expressed in *N. benthamiana* leaves (Figure 1c). The immunoblotting assay showed that VmEP1‐HA was present in the final GFP‐MdKRBP4‐precipitated immunocomplex (Figure 1c), indicating that MdKRBP4 interacts with VmEP1. Altogether, these results suggest that MdKRBP4 physically interacts with VmEP1.

### 2.2 Overexpression of *MdKRBP4* activates the immune response

**FIGURE 2:**
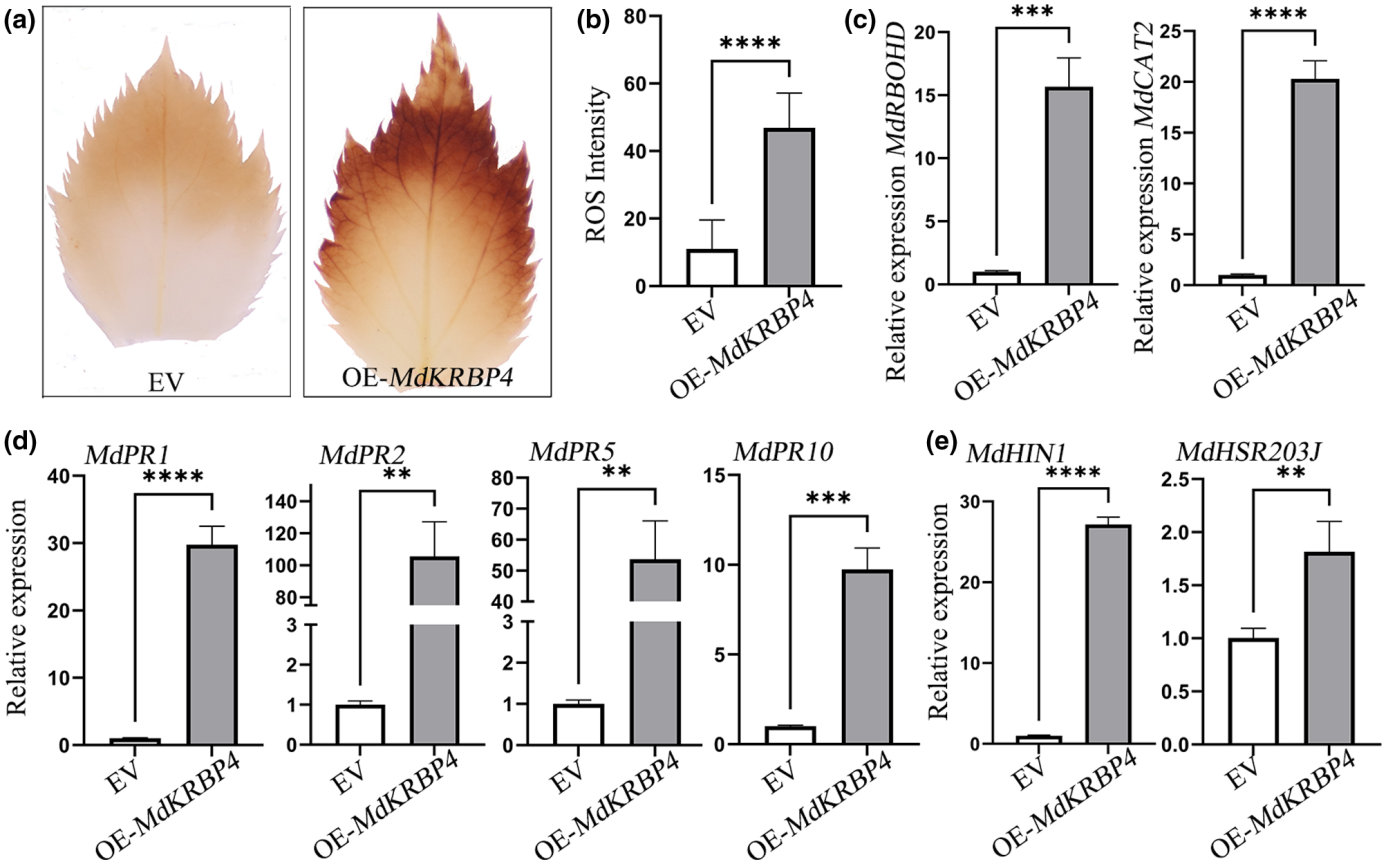
Overexpression of *MdKRBP4* activates a reactive oxygen species (ROS) burst. (a) 3,3′‐diaminobenzidine (DAB) staining shows ROS accumulation in apple leaves expressing *MdKRBP4*. (b) Quantification of ROS accumulation in apple leaves, as determined by ImageJ software (mean ± standard deviation; *n* > 10; ****p < 0.0001, Student's *t* test). (c) Relative transcript levels of ROS accumulation‐regulated key genes. (d) Relative transcript levels of marker genes of salicylic acid‐dependent immunity and *MdPR10* in apple leaves. (e) Relative transcript levels of hypersensitive response‐related genes. (c–e) These genes were analysed by reverse transcription‐quantitative PCR with *MdEF‐1α* as the internal reference. Apple leaves were infiltrated with *MdKRBP4* or empty vector (EV) control for 3 days. Relative transcript levels of genes were normalized to *MdEF‐1α* and calibrated to the levels of the EV control (set as 1) (mean ± standard deviation; *n* = 3; **p < 0.01, ***p < 0.001, ****p < 0.0001, Student's *t* test). These experiments were repeated three times with similar results.

Our studies revealed that VmEP1 targets MdKRBP4. Therefore, we speculated that MdKRBP4 is a vital factor regulating the immune response in plants, and we examined the accumulation of ROS using 3,3′‐diaminobenzidine (DAB) staining and mRNA accumulation of defence‐related genes (*PR1*, *PR2*, *PR5*) using reverse transcription‐quantitative PCR (RT‐qPCR) (Boutrot & Zipfel, 2017; Heath, 2000; Pontier et al., 1994; Zipfel & Oldroyd, 2017). Compared with controls, apple leaves overexpressing *MdKRBP4* showed high production of ROS, indicating a role of MdKRBP4 in inducing ROS accumulation (Figure 2a). Meanwhile, the expression levels of ROS accumulation‐regulating genes (Figure 2c) and salicylic acid pathway‐related genes, such as *MdPR1*, *MdPR2*, and *MdPR5* (Figure 2d), were considerably enhanced 3 days post‐infiltration. The relative transcript level of *MdPR10*, a target of VmEP1 (Wang et al., 2021), was also enhanced in apple leaves expressing *MdKRBP4* (Figure 2d). These results suggest that the transient expression of *MdKRBP4* activates the plant immune response and induces ROS accumulation.

### 2.3 MdKRBP4 induces cell death in *N. benthamiana* leaves

**FIGURE 3:**
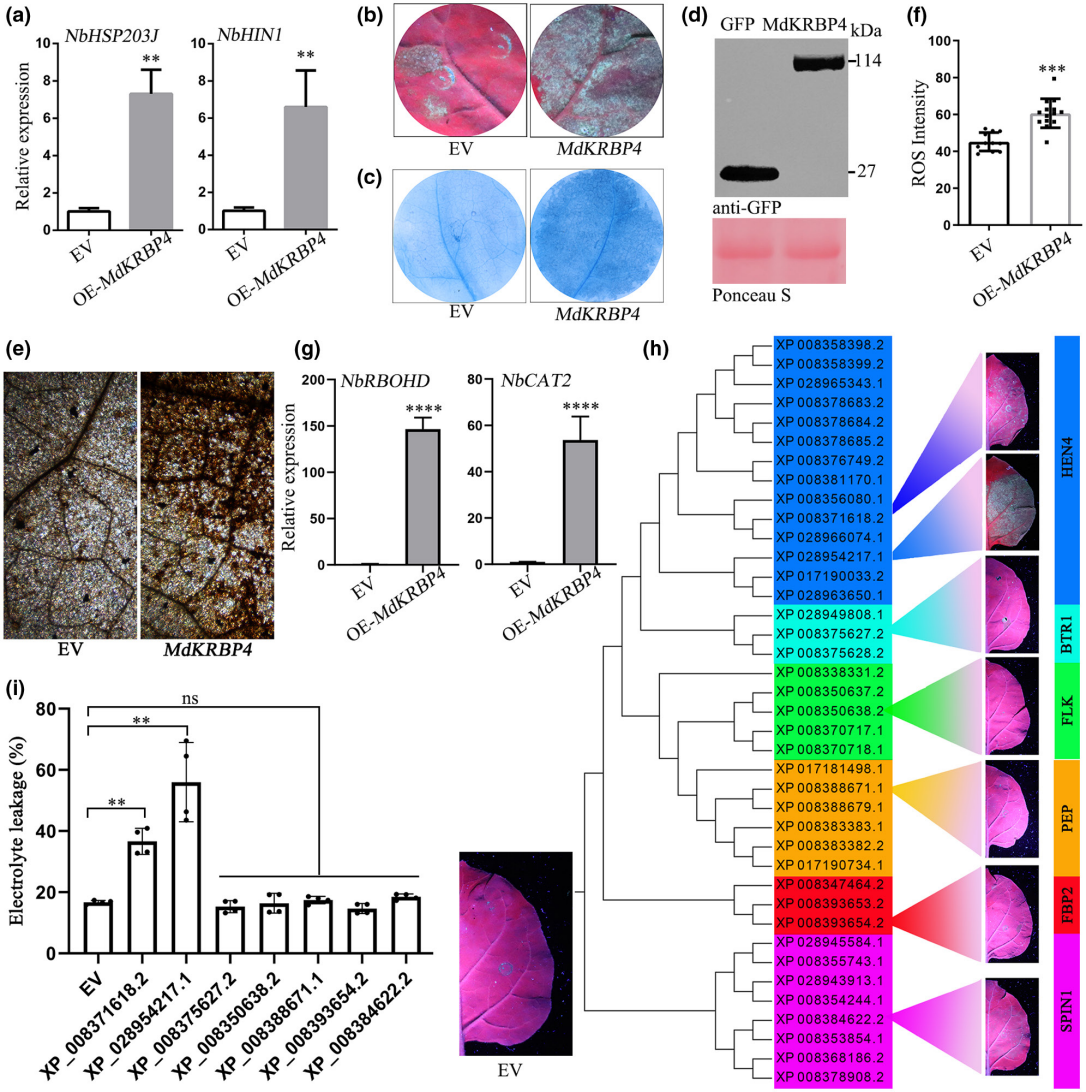
Overexpression of *MdKRBP4* induces cell death and the accumulation of reactive oxygen species (ROS) in *Nicotiana benthamiana* leaves. (a) Relative transcript levels of hypersensitive response‐related genes in *N. benthamiana* leaves expressing *GFP* or *MdKRBP4* were analysed by reverse transcription‐quantitative PCR (RT‐qPCR) with *NbActin*. *N. benthamiana* leaves expressing *MdKRBP4* or *GFP* control for 3 days were used for RNA extraction. Relative transcript levels of genes were normalized to *NbActin* and calibrated to the levels of the empty vector (EV) control (set as 1) (mean ± standard deviation; *n* = 3; **p < 0.01, Student's *t* test). Similar results were obtained from three individual experiments. (b) Photographs were taken at 5 days postinoculation under UV light. *N. benthamiana* leaves were transiently expressing EV (left) and *MdKRBP4* (right). (c) Trypan blue staining showing the cell death symptoms of the EV (left) and *MdKRBP4* (right) in leaves of *N. benthamiana*. (d) An anti‐GFP antibody was used to analyse expression of the marked constructs. (e) 3,3′‐diaminobenzidine (DAB) staining shows ROS accumulation in *N. benthamiana* leaves expressing *MdKRBP4*. (f) Quantification of ROS accumulation in *N. benthamiana* leaves, as determined by ImageJ software. Error bars indicate ± standard deviation, *n* > 10; ***p < 0.001, Student's *t* test. (g) The mRNA level of ROS accumulation‐regulated key genes in *N. benthamiana*, as revealed by RT‐qPCR 3 days postinfiltration (dpi) of overexpression construct OE‐*MdKRBP4*. The transcript levels were analysed by RT‐qPCR. Relative expression levels were normalized to *NbActin* and calibrated to the levels of the EV control (set as 1). Error bars indicate ± standard deviation, *n* = 3; ****p < 0.0001, Student's *t* test. These experiments were repeated three times with similar results. (h) Analysis of KH domain‐containing proteins in *Malus domestica*. The sequences were obtained from an *M. domestica* genome database by bioinformatics analysis. The tree was constructed with the maximum‐likelihood method. Coloured bars represent different subgroups. (i) Cell death was quantified by measuring electrolyte leakage (mean ± standard deviation; *n* = 4; **p < 0.01, Student's *t* test).

ROS are a critical signal that triggers and activates cell death in plants (Jacobson, 1996; Petrov & Van Breusegem, 2012). Therefore, we investigated whether MdKRBP4 induces cell death in apple leaves. Although RT‐qPCR analysis showed an up‐regulation of the hypersensitive response‐related genes *MdHSRP203J* (Pontier et al., 1998) and *MdHIN1* (Takahashi et al., 2004) (Figure 2e) a cell death phenotype in apple leaves expressing *MdKRBP4* was not detected. Interestingly, we found that *MdKRBP4* overexpression significantly up‐regulated the transcript levels of the hypersensitive response‐related genes *NbHSRP203J* and *NbHIN1* (Figure 3a) and induced cell death in *N. benthamiana* leaves (Figure 3b). Furthermore, staining with trypan blue confirmed that the lesions represented MdKRBP4‐induced cell death in *N. benthamiana* leaves (Figure 3c). Meanwhile, DAB staining showed that the transient expression of *MdKRBP4* promoted ROS accumulation in *N. benthamiana* compared to controls (Figure 3e,f). In addition, the RT‐qPCR analysis indicated that *MdKRBP4* up‐regulated the expression of *NbRBOHD* and *NbCAT2*, genes regulating ROS accumulation (Figure 3g). These results suggest that *MdKRBP4* induces ROS accumulation, resulting in cell death in *N. benthamiana*.

KH domain‐containing proteins possess a conserved VIGXXGXXI motif (Burd & Dreyfuss, 1994). We obtained VIGXXGXXI‐containing proteins from the apple genome database (ASM211411 v1). A total of 39 VIGXXGXXI‐containing proteins including MdKRBP4 (XP_028954217.1) were identified (Figure 3h). Phylogenetic tree analysis divided these proteins into six subgroups (Figure 3h). We randomly selected and cloned genes from each subgroup. Transient expression of XP_028954217.1 (*MdKRBP4*), XP_008371618.2, XP_008375627.2, XP_008350638.2, XP_008388671.1, XP_008393654.2, and XP_008384622.2 from each subgroup showed that only the genes from the HEN4 subgroup caused cell death in *N. benthamiana* (Figure 3h). In addition, *MdKRBP4* overexpression resulted in the highest electrolyte leakage, indicating maximum cell death (Figure 3i). These results imply that MdKRBP4 is essential for plant immunity.

### 2.4 MdKRBP4 positively regulates apple resistance to *V. mali*

**FIGURE 4:**
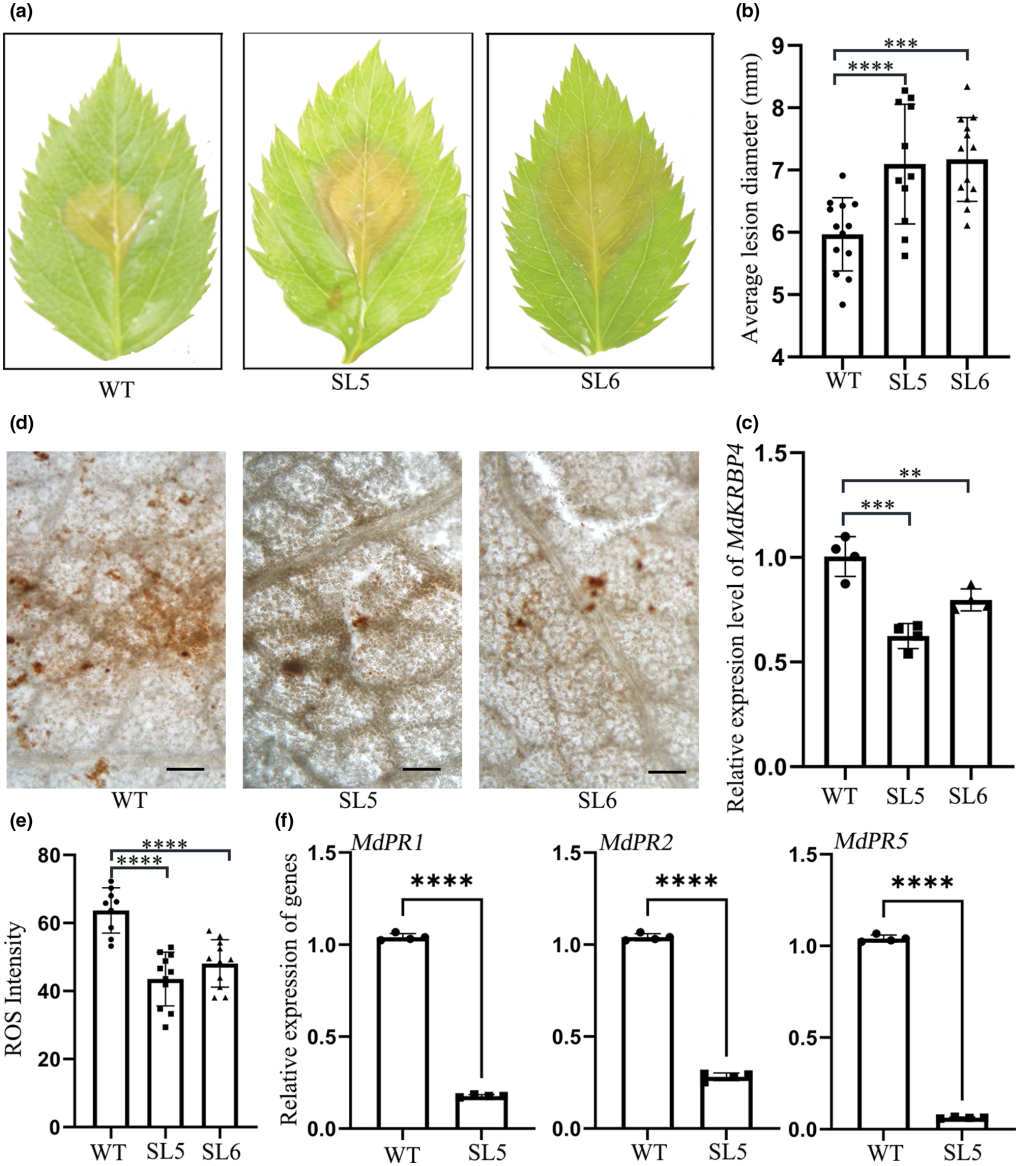
Silencing of *MdKRBP4* enhances sensitivity of apple to *Valsa mali*. (a) Silencing of *MdKRBP4* in apple leaves promotes *V. mali* infection. Wild‐type (WT) apple leaves and *MdKRBP4*‐silenced apple leaves (SL5, SL6) were inoculated with *V. mali*. Representative disease symptoms were photographed at 24 h postinoculation (hpi). (b) Average lesion diameters were measured at 24 hpi. Bars indicate ± standard deviation, *n* > 15; ***p < 0.001, ****p < 0.0001, Student's *t* test. These experiments were repeated three times with similar results. (c) Silencing efficiency of *MdKRBP4* in apple leaves was determined by reverse transcription‐quantitative PCR (RT‐qPCR) (mean ± standard deviation; *n* = 4; **p < 0.01, ***p < 0.001, Student's *t* test). These experiments were repeated three times with similar results. (d) 3,3′‐diaminobenzidine (DAB) staining shows reactive oxygen species (ROS) accumulation in apple leaves incubated with *V. mali*. Bar = 100 μm. (e) Quantification of ROS accumulation in apple leaves, as determined by ImageJ software. Means and standard deviation were calculated from three independent experiments. Bars indicate ± standard deviation; *n* > 15; ****p < 0.0001, Student's *t* test. (f) Relative transcript levels of *MdPR1*, *MdPR2*, and *MdPR5* in SL5 were analysed by RT‐qPCR with *MdEF‐1α*. Relative transcript levels of genes were normalized to *MdEF‐1α* and calibrated to the levels of the empty vector (EV) control (set as 1) (mean ± standard deviation; *n* = 4; ****p < 0.0001; Student's *t* test). Similar results were obtained from three individual experiments.

Subsequently, we constructed RNA interference vectors and transferred them to apple plants via *Agrobacterium*‐mediated transformation to investigate whether MdKRBP4 positively regulates immunity. After RT‐qPCR evaluation, we obtained two silencing lines (SL5 and SL6) in which *MdKRBP4* expression was less than 35% of that in wild‐type (WT) plants (Figure 4c). Then, we infected WT, SL5, and SL6 plants with *V. mali*. The SL5 and SL6 plants were more susceptible than the WT (Figure 4a,b). In addition, ROS accumulation in SL5 and SL6 apple leaves inoculated with *V. mali* was obviously less than that in inoculated WT apple leaves (Figure 4d,e), indicating that silencing of *MdKRBP4* weakened ROS generation. We also found that silencing of *MdKRBP4* down‐regulated the expression of *MdPR1*, *MdPR2*, and *MdPR5* (Figure 4f). These results suggest that MdKRBP4 positively regulates apple resistance to *V. mali*.

### 2.5 VmEP1 inhibits plant immunity induced by MdKRBP4

**FIGURE 5:**
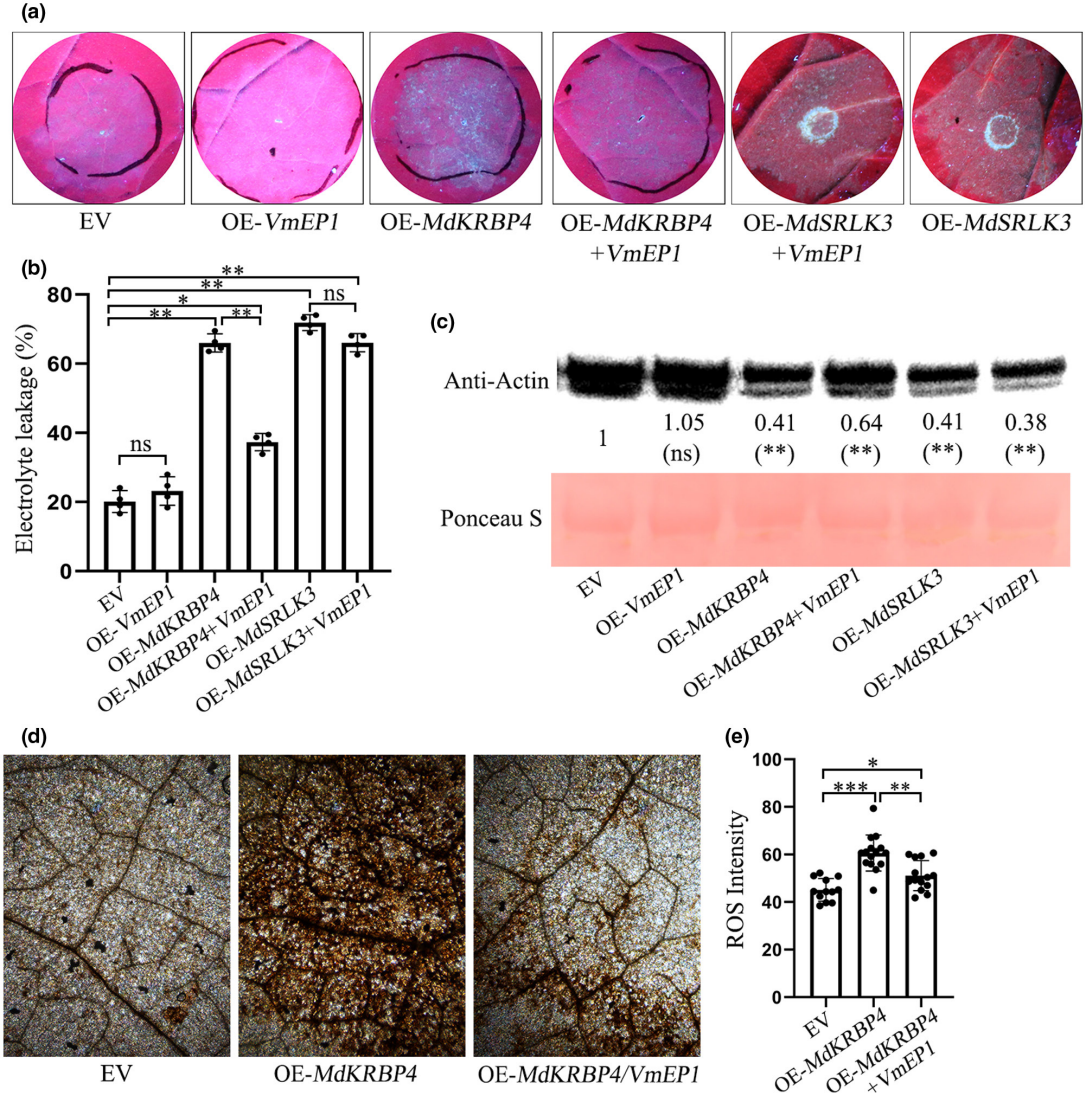
VmEP1 inhibits cell death induced by *MdKRBP4* in *Nicotiana benthamiana*. (a) Leaves of *N. benthamiana* were infiltrated with *Agrobacterium tumefaciens* carrying pBICAM1302 (empty vector [EV]), *VmEP1*, *MdKRBP4*, and *MdKRBP4*/*VmEP1. MdSRLK3* and *MdSRLK3*/*VmEP1* were used as controls. Photographs were taken at 5 days postinfiltration (dpi) under UV light. (b) Quantification of cell death by measuring electrolyte leakage. (c) Actin levels in *N. benthamiana* leaves expressing *GFP*, *VmEP1*, *MdKRBP4*, *MdKRBP4*/*VmEP1*, *MdSRLK3*, and *MdSRLK3*/*VmEP1* were analysed by western blot, showing the total protein extracted from the same amount of leaves in different samples at 5 dpi. Asterisks indicate significant differences (**p < 0.01 compared with GFP expression, Student's *t* test). This experiment was repeated three times with similar results. (d) 3,3′‐diaminobenzidine (DAB) staining shows reactive oxygen species (ROS) accumulation in *N. benthamiana* leaves expressing EV, *MdKRBP4*, or *MdKRBP4*/*VmEP1*. (e) Quantification of ROS accumulation in *N. benthamiana* leaves, as determined by ImageJ software. Means and standard deviation were calculated from three biological repeats. (b,e) Bars indicate ± standard deviation. *p < 0.05, **p < 0.01, ***p < 0.001; Student's *t* test.

We further tested whether VmEP1 affects MdKRBP4‐induced cell death and ROS accumulation. The result showed that VmEP1 attenuated MdKRBP4‐induced cell death in *N. benthamiana* (Figure 5a). Moreover, MdKRBP4‐triggered electrolyte leakage in *N. benthamiana* was significantly attenuated in the presence of VmEP1 compared with controls (Figure 5b). The analysis of actin quantity showed that the quantity of actin in *N. benthamiana* leaves expressing *MdKRBP4* was lower than that in *N. benthamiana* leaves expressing *MdKRBP4*/*VmEP1* (Figure 5c). We then conducted DAB staining to investigate the variations in ROS accumulation induced by MdKRBP4 in the presence of VmEP1. ROS accumulation in leaves coexpressing *MdKRBP4*/*VmEP1* was lower than that in leaves expressing *MdKRBP4* alone (Figure 5d,e). Previously, Yin demonstrated that MdSRLK3, a G‐type lectin S‐receptor‐like protein kinase from apple, induces cell death in *N. benthamiana* (Yin, 2018). Therefore, we used the coexpression of *MdSRLK3* and *VmEP1* as a control. The results showed that MdSRLK3‐induced cell death was not affected by VmEP1 (Figure 5a), indicating a specific effect of VmEP1 on MdKRBP4. Collectively, these results suggest that VmEP1 inhibits host immunity by targeting MdKRBP4 and attenuating the immune response triggered by MdKRBP4.

### 2.6 MdKRBP4 is a virulence target of VmEP1

**FIGURE 6:**
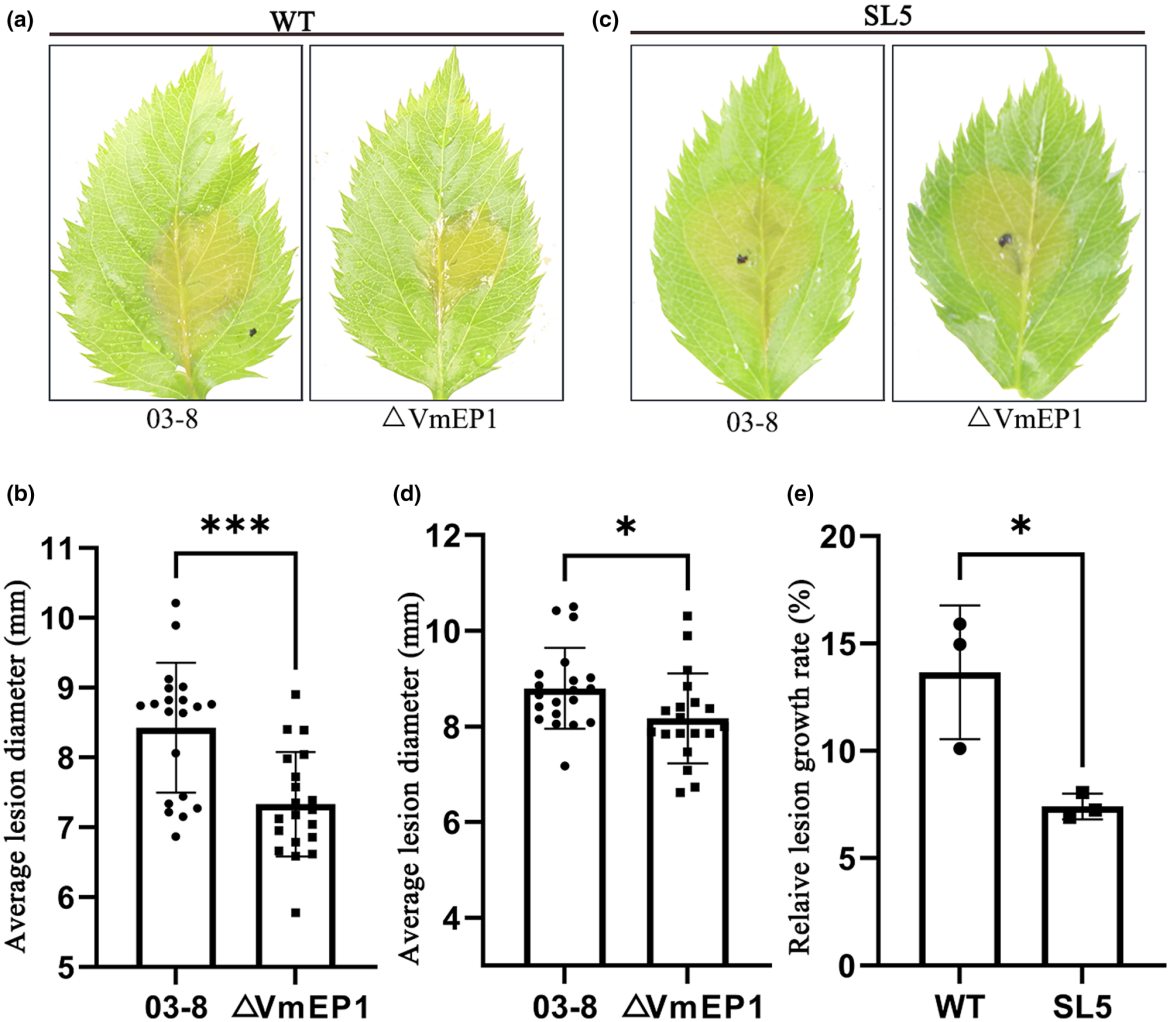
MdKRBP4 is essential for the virulence of VmEP1. (a) Wild‐type (WT) apple leaves were inoculated with WT *Valsa mali* and ΔVmEP1. (c) Transgenic SL5 apple leaves were inoculated with *V. mali* and ΔVmEP1. (b,d) Average lesion diameters were measured at 24 h postinfection (hpi). Bars indicate ± standard deviation, *n* > 15; ***p < 0.001, *p < 0.05, Student's *t* test. These experiments were repeated three times with similar results. (e) Relative lesion growth rate of WT *V. mali* and ΔVmEP1. The relative lesion growth rate was calculated as follows: relative lesion growth rate (%) = (average lesion diameter of WT *V. mali* − average lesion diameter of ΔVmEP1) × 100/(average lesion diameter of ΔVmEP1). Means and standard deviations were calculated from three independent experiments. Bars indicate ± standard deviation. *p < 0.05, Student's *t* test.

Furthermore, we verified whether MdKRBP4 is a virulence target of VmEP1. A VmEP1 deletion mutant (ΔVmEP1) (Li et al., 2015) and WT *V. mali* were inoculated on WT apple leaves and SL5 leaves. The average lesion diameter of WT *V. mali* was smaller than that of ΔVmEP1 in WT apple leaves (Figure 6a,b). The average lesion diameter of WT *V. mali* was also smaller than that of ΔVmEP1 in SL5 leaves (Figure 6c,d). However, the lesion growth rate on SL5 leaves was slower compared to the lesion growth rate on WT leaves (Figure 6e), indicating that MdKRBP4 is required for the virulence function of VmEP1. These results suggest that MdKRBP4 is essential for the virulence of VmEP1.

### 2.7 VmEP1 promotes MdKRBP4 degradation and inhibits *MdPR10* expression

**FIGURE 7:**
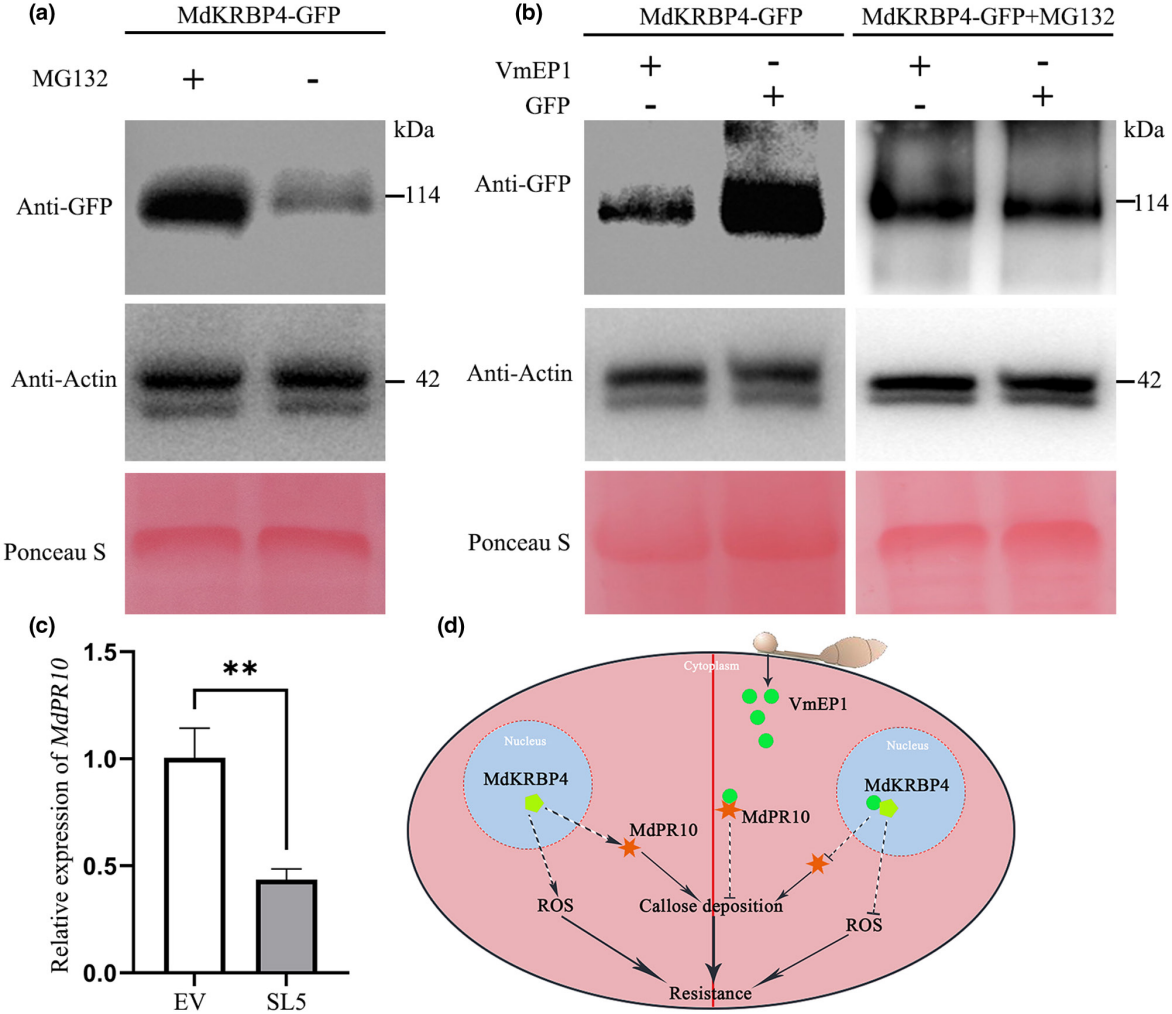
VmEP1 inhibits the expression of *MdPR10* by decreasing the accumulation of MdKRBP4. (a) Analysis of the protein stability of MdKRBP4 by western blot. *Nicotiana benthamiana* leaves expressing *MdKRBP4* were treated with 40 μM MG132 for 30 min before extracting total protein. Anti‐GFP antibodies were used to detect MdKRBP4. Anti‐actin was used as loading control. Membranes were stained with Ponceau S to verify equal loading. This experiment was repeated three times. (b) The effect of VmEP1 on MdKRBP4 on protein stability was analysed by western blot. Total protein was extracted from *N. benthamiana* leaves expressing *MdKRBP4‐GFP*/*VmEP1‐HA* or *MdKRBP4‐GFP*/*GFP* and immunoblotted using anti‐GFP to detect MdKRBP4 (left). *N. benthamiana* leaves were treated with 40 μM MG132 for 30 min before extracting total protein. Anti‐GFP antibodies were used to detect MdKRBP4 (right). Anti‐actin was used as loading control. Membranes were stained with Ponceau S to verify equal loading. This experiment was repeated three times. (c) Relative transcript levels of *MdPR10* in SL5 apple were analysed by reverse transcription‐quantitative PCR  with *MdEF‐1α* as the internal reference (mean ± standard deviation; *n* = 3; **p < 0.01, Student's *t* test). The experiment was repeated three times with similar results. (d) A working model for the interaction between VmEP1 and MdKRBP4. Without *Valsa mali* infection, MdKRBP4 enhances apple resistance by indirectly up‐regulating *MdPR10* and reactive oxygen species (ROS) levels. Upon *V. mali* infection, the effector VmEP1 is secreted into the nucleus to interact with MdKRBP4, which leads to the degradation of MdKRBP4. The degradation of MdKRBP4 indirectly results in down‐regulation of *MdPR10* and ROS levels, reducing resistance to *V. mali*.

Immunoprecipitation (IP) previously revealed that the MdKRBP4 protein is easily degraded (Dharmasiri et al., 2005). The peptide‐aldehyde proteasome inhibitor MG132 suppressed MdKRBP4 degradation (Figure 7a), indicating that MdKRBP4 is degraded by the 26S proteasome pathway in plants (Dreher & Callis, 2007). Initial experiments proved that VmEP1 mitigated ROS production and cell death induced by MdKRBP4 (Figure 3); therefore, we tested whether VmEP1 decreases the MdKRBP4 protein level. We measured the MdKRBP4 protein level after coexpression of *MdKRBP4* with *VmEP1* in *N. benthamiana* leaves. Agrobacteria containing either *MdKRBP4‐GFP* or *GFP* were coinfiltrated with *VmEP1‐HA*, and leaves were collected at 48 h postinoculation (hpi). As exhibited in Figure 7b, lower fluorescence of MdKRBP4‐GFP was detected in the presence of VmEP1‐HA than that of GFP in the presence of VmEP1‐HA (Figure 7b, left), suggesting that the transient expression of *VmEP1* decreases MdKRBP4 protein accumulation. Interestingly, MG132 treatment blocked the degradation of MdKRBP4 by VmEP1 (Figure 7b, right). However, the interaction between VmEP1 and MdPR10, a target protein of VmEP1 (Wang et al., 2021), did not result in degradation of MdPR10 (Figure S2), indicating that VmEP1 specifically promotes MdKRBP4 degradation. These results indicate that the interaction between VmEP1 and MdKRBP4 reduces MdKRBP4 accumulation by promoting its degradation.

Previous studies have shown that VmEP1 interacts with MdPR10, an immune‐related protein (Wang et al., 2021). In the present study, we found that VmEP1 targets MdKRBP4 and regulates the plant immune response. Overexpression of *MdKRBP4* increased the relative expression level of *MdPR10* (Figure 2d). Coincidentally, the relative expression level of *MdPR10* was reduced in SL5, indicating that MdKRBP4 indirectly regulates *MdPR10* expression (Figure 7c). Our results show that MdKRBP4, as a positive regulator of plant immunity, enhances apple resistance to *V. mali* by up‐regulating *MdPR10* expression and ROS accumulation. VmEP1 secreted by *V. mali* interacts with MdKRBP4 in the nucleus, reducing the accumulation of MdKRBP4. The decrease in MdKRBP4 indirectly down‐regulates *MdPR10* expression and ROS accumulation, reducing apple resistance to *V. mali* (Figure 7d).

## 3 DISCUSSION

Research has shown that KH domain‐containing proteins such as PEPPER, SPIN1, FLK, and HEN4 regulate the development of *A. thaliana* (Cheng et al., 2003; Lim et al., 2004; Ripoll et al., 2009; Vega‐Sanchez et al., 2008). In the present study we chose PEPPER, SPIN1, FLK, and HEN4 from Cruciferae, Gramineae, Musaceae, Celastraceae, Rosaceae, and Malvaceae and constructed a phylogenetic tree using ClustalW. The results show that KH domain‐containing proteins regulating plant development can be grouped into four distinct classes (Figure S3). AtHEN4 and MdKRBP4 were within the same subgroup, and multiple sequence alignment of the HEN4 subgroup indicated that they had a KH domain (Figure S4). Subsequently, we identified eight genes (Table S2) as putative apple HEN4‐encoding genes via BLASTP search against the apple proteome using the characterized AtHEN4, which delays flowering (Cheng et al., 2003; Ortuno‐Miquel et al., 2019). This observation implied that the apple genes might also control flowering. However, no evidence indicated that HEN4 participates in the plant immune response. Remarkably, in the present study we found that silencing of *MdKRBP4* increased the susceptibility of apple to *V. mali* (Figure 4), indicating MdKRBP4 positively regulates the plant immune response. Overexpression of *MdKRBP4* induced an ROS burst, indicating that MdKRBP4 participates in the plant immune response by regulating ROS levels.

ROS, such as the superoxide anion (O_2_•^−^) and hydrogen peroxide (H_2_O_2_), play an important role in resisting infection of pathogens during the plant defence response (Shetty et al., 2008). However, pathogens differ in their sensitivities to ROS. For instance, 0.1 mM H_2_O_2_ suppresses the infection of *Pectobacterium carotovorum* and *Phytophthora infestans* (Wu et al., 1995). ROS are toxic to plants. Studies have revealed that a regulated increase in ROS benefits cell differentiation and proliferation (Schafer & Buettner, 2001), while an excess leads to a series of physiological changes, such as nucleic acid degradation, lipid peroxidation, and enzyme inactivation (Lamb & Dixon, 1997; Trachootham et al., 2009). For example, 6–8 mM H_2_O_2_ leads to the death of suspension‐cultured soybean cells (Levine et al., 1994). Interestingly, in the present study we detected cell death (Figure 5a) and abundant accumulation of ROS (Figure 5g) in *N. benthamiana* leaves overexpressing *MdKRBP4*. However, we only observed ROS accumulation, but no cell death, in *M. domestica* leaves (Figure 2a). Previous studies demonstrated that different plants can tolerate different concentrations of H_2_O_2_, such as 60 μM–7 mM in *Arabidopsis* and 1–2 mM in maize and rice (Wang et al., 2013). Based on the above studies, we speculate that perhaps *N. benthamiana* leaves are more sensitive to H_2_O_2_ than *M. domestica* leaves, which leads to cell death in *N. benthamiana* leaves. Another possibility is that MdKRBP4‐induced ROS accumulation in *N. benthamiana* leaves is greater than in *M. domestica* leaves, and therefore cell death was not observed in apple leaves expressing *MdKRBP4*.

Biotic and abiotic stresses lead to ROS accumulation. Rapid production of ROS is a typical characteristic of the hypersensitive response following the recognition of pathogen infection (Lamb & Dixon, 1997; Wojtaszek, 1997). Pathogens adopt measures to inhibit ROS production. For example, the *Puccinia striiformis* effector Pst18363 can target and stabilize wheat Nudix hydrolase 23 (TaNUDX23) to suppress ROS accumulation and facilitate infection (Yang et al., 2020). In the present study we detected ROS production in *MdKRBP4‐*expressing leaves of apple (Figure 2) and *N. benthamiana* (Figure 5d). Furthermore, stable silencing of *MdKRBP4* decreased *V. mali*‐induced ROS accumulation (Figure 4d). These results indicate that MdKRBP4 modulates ROS production to positively regulate the plant immune response. We also found that the *V. mali* effector VmEP1 inhibited cell death (Figure 5a) and decreased MdKRBP4‐induced ROS accumulation in *N. benthamiana* (Figure 5c). BiFC and Co‐IP assays demonstrated the interaction between VmEP1 and MdKRBP4 (Figure 1). These results indicate that VmEP1 promotes *V. mali* infection by restraining MdKRBP4‐induced ROS accumulation.

Pathogens use effectors as key weapons to attack host plants because they can manipulate plant immunity by disrupting host protein function and promoting infection. For example, *Pseudomonas syringae* HopZ1a suppresses local and systemic plant immunity by acetylating mitogen‐activated protein kinase kinase 7 (MKK7) (Rufian et al., 2021). The *Magnaporthe oryzae* effector AvrPiz‐t suppresses the ubiquitin ligase activity of the rice RING E3 ubiquitin ligase APIP6 to suppress PAMP‐triggered immunity (Park et al., 2012). AVR3a of *P. infestans* restrains host cell death by targeting and stabilizing host U‐box E3 ligase CMPG1 during the biotrophic phase of infection (Bos et al., 2010), and Avr1d from *Phytophthora sojae* enhances the stability of the soybean E3 ubiquitin ligase GmPUB13 to facilitate infection (Lin et al., 2021). These studies illustrated that effectors control host immunity in various ways, especially manipulating host protein ubiquitination. In the present study we found that the proteasome inhibitor MG132 stabilized MdKRBP4 (Figure 7a), suggesting that the MdKRBP4 protein is degraded via the 26S proteasome pathway. We demonstrated that VmEP1 targets MdKRBP4 (Figure 1), leading to MdKRBP4 degradation (Figure 7a). The ubiquitin E3 ligase PUB17 degrades a KH domain‐containing protein, StKH17 (McLellan et al., 2020). These results imply that VmEP1 restrains ROS accumulation and promotes *V. mali* infection by disrupting MdKRBP4 ubiquitination.

Our findings prove that MdKRBP4 has a novel function in plant immunity. We found that the VmEP1 effector promotes the degradation of the positive immune regulator MdKRBP4 to compromise plant defence. These findings provide a basis for detailed research on apple resistance. However, additional studies are necessary to elucidate the MdKRBP4‐mediated immune regulatory mechanism.

## 4 EXPERIMENTAL PROCEDURES

### 4.1 Plant and microbe materials

The tissue‐cultured plantlets of *M. domestica* ‘Gala’ and *MdKRBP4*‐silenced transgenic plants were initially grown for 4 weeks on Murashige and Skoog (MS) agar supplemented with 0.3 mg/L 6‐benzylaminopurine and 0.2 mg/L indole‐3‐acetic acid. They were cultured at 25°C under a 14/10‐h light/dark photoperiod with a light intensity of 60 μmol·m^−2^·s^−1^. *N. benthamiana* plantlets were cultured in an incubator under a 16/8‐h light/dark photoperiod at 25°C/22°C and used for *Agrobacterium* infiltration experiments.

*V. mali* was acquired from the State Key Laboratory of Crop Stress Biology for Arid Areas, Northwest A&F University, Shaanxi, Yangling, China and cultured on potato dextrose agar at 25°C.

### 4.2 Transient expression of *MdKRBP4* in *N. benthamiana* and *M. domestica*

The transient expression constructs were generated with the plant expression vector pCAMBIA1302 using standard protocols. The vectors were then transformed into *A. tumefaciens* GV3101 (pSoup‐P19) by electroporation. The *Agrobacterium* strains containing constructs were cultured in yeast extract peptone broth in a shaker at 200 rpm at 28°C for 18 h. The bacteria were harvested in infiltration buffer (10 mM MgCl_2_, 10 mM MES, and 200 μM acetosyringone, pH 5.7) and incubated for 3 h without light at room temperature before use. Five‐week‐old *N. benthamiana* leaves were infused with the binary constructs using a 1‐ml syringe without a needle.

The infiltration of apple leaves was carried out as previously described (Wang et al., 2021). Briefly, *Agrobacterium* suspensions were used to prepare 50 ml *Agrobacterium* containing the pCAMBIA1302 constructs, which was then vacuum‐infiltrated into apple seedling leaves under 100 kPa for 10 min. The treated seedlings were cultured on MS medium for 2 days and then used for experiments.

### 4.3 Y2H assay

*MdKRBP4* and *VmEP1* were cloned into the binary vectors pGADT7 and pGBDT7, respectively. Primers used in this study are summarized in Table S3. The polyethylene glycol‐mediated conversion method stated in the Yeast Protocols Handbook (Clontech) was used to transform binary vectors into *Saccharomyces cerevisiae* AH109. pGADT7‐MdKRBP4 and pGBDT7‐VmEP1 were cotransformed into yeast cells, and then transformants were selected on synthetic dropout (SD) medium without tryptophan (Trp) and leucine (Leu). Then, single clones were transferred onto SD medium lacking adenine (Ade), histidine (His), Leu, and Trp, containing X‐α‐Gal for selection of interaction.

### 4.4 BiFC assay

*MdKRBP4* and *VmEP1* were cloned into the binary vectors cYFP and nYFP, respectively. Table S3 contains the information of primers applied in the present study. The obtained vectors were transformed into *A. tumefaciens* GV3101 and coexpressed in *N. benthamiana* leaves. A confocal laser scanning microscope (FV3000; Olympus) was used to detect the results at about 48 hpi.

### 4.5 Co‐IP assay

Co‐IP assays were executed to verify the protein interactions in vivo. To construct the MdKRBP4‐GFP vector, *MdKRBP4* was cloned into pCAMBIA1302 via homologous recombination, resulting in pCAMBIA1302‐*MdKRBP4*, in which the expression of the VmEP1‐GFP fusion is driven by a CaMV 35S promoter. VmEP1 was fused into the vector PICH86988 with an HA tag at its C‐terminus. Table S3 contains the information of primers applied in the present study. All plasmids were transformed into *A. tumefaciens* GV3101 and expressed in *N. benthamiana* leaves by coinfiltration. After infiltration with *A. tumefaciens* with either VmEP1‐GFP or GFP, *N. benthamiana* leaf tissues were collected at about 36 hpi and frozen in liquid nitrogen. The proteins were extracted in native lysis buffer containing 1% protease inhibitor cocktail and 1 mM phenylmethylsulfonyl fluoride (Solarbio). The protein extracts were cleaned by two rounds of centrifugation at 15,000 × *g* for 10 min to remove tissue debris. Next, the total proteins were incubated with 20 μl anti‐GFP magnetic beads (Epizyme) for 3 h at 4°C. Then, the magnetic beads were washed three times with TBST buffer (50 mM Tris–HCl, pH 7.5, 8 g/L NaCl, 0.2 g/L polysorbate 20) to elute the proteins, which were boiled in 1× SDS‐PAGE loading buffer for 10 min. The proteins were separated by 10% SDS‐PAGE and examined by western blot with anti‐GFP antibody (Abmart, M20004, 1:5000 dilution) or anti‐HA antibody (Abmart, M20003, 1:3000 dilution). Antibody binding was detected using chemiluminescent horseradish peroxidase substrate (Millipore). GFP IP experiments were carried out with an anti‐GFP magnetic beads kit (Epizyme) following the manufacturer's instructions.

### 4.6 IP and mass spectrometry analysis

For IP and mass spectrometry, the sequence of VmEP1 was inserted into the pCAMBIA1302 vector fused with GFP. Table S3 contains the information of primers used in this study. The total proteins of leaves expressing *VmEP1* were extracted and purified in native lysis buffer. The total protein mixture was centrifuged at 14,000 × *g* for 10 min and tissue debris was discarded. Next, 30 μl anti‐GFP magnetic beads (Shanghai Epizyme Biomedical Technology) was mixed into the supernatant and samples were incubated for 3 h at 4°C on a rotator. The anti‐GFP magnetic beads were washed about five times using TBST and the beads were boiled with 1× SDS‐PAGE loading buffer for 10 min. The total proteins were separated by 10% SDS‐PAGE. The gel was then stained using a kit from Thermo (MAN0011539) following the manufacturer's instructions. After staining with silver stain and subsequent destaining, gel sections were excised and then digested with trypsin. Finally, mass spectrometry was conducted to identify interacting proteins as previously described (Hu et al., 2014).

### 4.7 Construction of transgenic apple

RNA interference vectors for apple were generated using the plant expression vector pK7GWIWG2D. The specific PCR fragments of *MdKRBP4* were inserted into pK7GWIWG2D(II). Table S3 contains the information of primers applied in the present study. *Agrobacterium‐*mediated transformation was used for transforming apples (Dai et al., 2013). Briefly, 50 ml *Agrobacterium* suspension was prepared in which leaf segments were shaken gently for 5–8 min. Excess fluid was wiped off with sterile filter paper. These leaf segments were then cocultivated on MS agar containing 2 mg/ml thidiazuron (TDZ), 0.5 mg/ml indole‐3‐butyric acid (IBA), and 100 μM acetosyringone in dark conditions for 2 days. Immediately after cocultivation, explants were transferred to MS agar with 2 mg/L TDZ, 0.5 mg/L IBA, and 250 mg/L Timentin in the dark for 3 days. Explants were then transferred to MS agar with 2 mg/L TDZ, 0.5 mg/L IBA, 250 mg/L Timentin, and 25 mg/L kanamycin in the dark for about 21 days of selection culture. Then, kanamycin‐resistant buds were transferred to MS agar with 2 mg/L TDZ, 0.5 mg/L IBA, 250 mg/L Timentin, and 25 mg/L kanamycin in the light. The transgenic seedlings were moved to fresh medium every 2 weeks to maintain selection pressure.

### 4.8 Pathogen inoculation

*V. mali* (03–8) was cultured on potato dextrose agar for 2 days at 28°C. Plugs (3 mm diameter) of *V. mali* were incubated onto transgenic apple leaves for about 24 h at 28°C using the stab inoculation method. Resulting lesions were photographed and the size was evaluated by ImageJ. Each of the experiments was done on at least 30 apple seedling leaves, and for all inoculation assays three biological repeats were carried out.

### 4.9 Isolation of RNA and RT‐qPCR

RNA was isolated from samples (50 mg) using the EasyPure Plant RNA kit (Transgen) and processed with DNase I. RNA samples (2 μg) were reverse‐transcribed using high‐capacity cDNA reverse transcription kits (Applied Biosystems) and subjected to qPCR using gene‐specific primers (available upon request). cDNA samples were amplified using 2× RealStar Green Power mixture (GenStar) and a Roche LightCycler 96 SW1.1 real‐time PCR system (Roche). The elongation factor 1α (*EF‐1α*) gene of *M. domestica* and *Actin* of *N. benthamiana* were used for normalization, and gene expression was calculated by the comparative *C*_t_ method. Primers used in this study are summarized in Table S3. All experiments were repeated independently three times.

### 4.10 DAB staining

H_2_O_2_ accumulation in plant tissue was examined by staining with DAB as described previously (Xiao et al., 2003). Leaf pieces were infiltrated in DAB solution (Sigma) (1 mg/ml, pH 3.8) and shaken at room temperature for 12 h in the light. The leaf pieces were then destained with 95% ethanol. The cleared leaves were transferred to 50% glycerol. A microscope (Olympus) was used to take photographs. The accumulation of ROS was evaluated by ImageJ.

### 4.11 Trypan blue staining

*N. benthamiana* leaves expressing *MdKRBP4* or empty vector were stained by boiling for 10 min in lactophenol–trypan blue solution (10 ml lactic acid, 10 ml glycerol, 10 g phenol, 10 mg trypan blue, 10 ml distilled water). Then, they were decoloured with gentle shaking in a chloral hydrate solution (2.5 g/ml) for 12 h. Samples were photographed under natural light.

### 4.12 Electrolyte leakage

Cell death was quantified by determining electrolyte leakage using a previously described method (Ma et al., 2021; Nayyar & Chander, 2004). Samples from *N. benthamiana* (diameter 1 cm) expressing *MdKRBP4* were immersed in nanopure water (5 ml) for 3 h at room temperature to determine the electrical conductivity (E_1_). A conductivity meter (Five Easy Plus Conductivity) was used to measure the conductivity. Then the samples were boiled for 10 min and the second electrical conductivity (E_2_) was measured after the fluid recovered to ambient temperature. Electrolyte leakage was calculated as follows: electrolyte leakage (%) = (E_1_/E_2_) × 100. This assay was repeated three times.

### 4.13 Bioinformatics analysis

Multiple sequence alignment of HEN4 was performed with Clustal Omega (https://www.ebi.ac.uk/Tools/msa/). Phylogenetic trees were established using MEGA 7 software with the neighbour‐joining method (Kumar et al., 2016).

## AUTHOR CONTRIBUTIONS

W.W. and L.H. conceived the research. W.W. mainly performed the experiments. W.S., W.G., and L.L. assisted with the rest of the experiments. J.N. and L.X. participated in the preparation of the manuscript. L.H. revised the manuscript.

## CONFLICT OF INTEREST

The authors of this paper declare no competing interests exist.

## Supporting information


**FIGURE S1** Expression of *VmEP1* and *MdKRBP4* in plants. (a) Subcellular localization of VmEP1 was visualized by confocal microscopy in *Nicotiana benthamiana*. (b) Subcellular localization of MdKRBP4 was visualized by confocal microscopy in *N. benthamiana*. Scale bar represents 40 μm

**FIGURE S2** The effect of VmEP1 on MdPR10 protein accumulation was analysed by western blot. Total protein was extracted from *Nicotiana benthamiana* leaves expressing *MdPR10‐GFP*/*VmEP1‐HA* or *MdPR10‐GFP*/*GFP* and immunoblotted using anti‐GFP to detect MdPR10. Anti‐actin and Coomassie brilliant blue (CBB) staining were used as loading control

**FIGURE S3** Phylogenetic tree constructed using amino acid sequences of KH domain‐containing proteins. Shown is the phylogeny of MdKRBP4 and its homologous sequences from selected species including Cruciferae (*Arabidopsis thaliana*), Gramineae (*Triticum dicoccoides*, *Zea mays*, *Oryza sativa*, *Sorghum bicolor*), Musaceae (*Musa acuminata*), Celastraceae (*Tripterygium wilfordii*), Rosaceae (*Malus domestica*), and Malvaceae (*Gossypium hirsutum*). The tree was constructed with the neighbour‐joining method

**FIGURE S4** Multiple alignment of HEN4 sequences. The amino acid sequences were aligned by Clustal Omega, and conserved GXXG motifs are highlighted in different colours

**TABLE S1** NCBI BLASTp results of AtHEN4 in *Malus domestica* (taxid:3750)

**TABLE S2** Primers used in this study

**TABLE S3** PCR primers used in this study

## Data Availability

The data used in this study are available from the corresponding author.

## References

[mpp13248-bib-0001] Boevink, P.C. , Wang, X. , McLellan, H. , He, Q. , Naqvi, S. , Armstrong, M.R. et al. (2016) A *Phytophthora infestans* RXLR effector targets plant PP1c isoforms that promote late blight disease. Nature Communications, 7, 10311. 10.1038/ncomms10311 PMC4740116 26822079

[mpp13248-bib-0002] Boller, T. & Felix, G. (2009) A renaissance of elicitors: perception of microbe‐associated molecular patterns and danger signals by pattern‐recognition receptors. Annual Review of Plant Biology, 60, 379–406. 10.1146/annurev.arplant.57.032905.105346 19400727

[mpp13248-bib-0003] Bos, J.I. , Armstrong, M.R. , Gilroy, E.M. , Boevink, P.C. , Hein, I. , Taylor, R.M. et al. (2010) *Phytophthora infestans* effector AVR3a is essential for virulence and manipulates plant immunity by stabilizing host E3 ligase CMPG1. Proceedings of the National Academy of Sciences of the United States of America, 107, 9909–9914. 20457921 10.1073/pnas.0914408107 PMC2906857

[mpp13248-bib-0004] Boutrot, F. & Zipfel, C. (2017) Function, discovery, and exploitation of plant pattern recognition receptors for broad‐spectrum disease resistance. Annual Review of Phytopathology, 55, 257–286. 10.1146/annurev-phyto-080614-120106 28617654

[mpp13248-bib-0005] Burd, C.G. & Dreyfuss, G. (1994) Conserved structures and diversity of functions of RNA‐binding proteins. Science, 265, 615–621. 8036511 10.1126/science.8036511

[mpp13248-bib-0006] Chen, T. , Cui, P. , Chen, H. , Ali, S. , Zhang, S. & Xiong, L. (2013) A KH‐domain RNA‐binding protein interacts with FIERY2/CTD phosphatase‐like 1 and splicing factors and is important for pre‐mRNA splicing in *Arabidopsis* . PLoS Genetics, 9, e1003875. 24146632 10.1371/journal.pgen.1003875 PMC3798263

[mpp13248-bib-0007] Cheng, Y. , Kato, N. , Wang, W. , Li, J. & Chen, X. (2003) Two RNA binding proteins, HEN4 and HUA1, act in the processing of AGAMOUS pre‐mRNA in *Arabidopsis thaliana* . Developmental Cell, 4, 53–66. 12530963 10.1016/s1534-5807(02)00399-4 PMC5135010

[mpp13248-bib-0008] Cheng, Y.T. , Zhang, L. & He, S.Y. (2019) Plant‐microbe interactions facing environmental challenge. Cell Host & Microbe, 26, 183–192. 31415751 10.1016/j.chom.2019.07.009 PMC6697056

[mpp13248-bib-0009] Dai, H. , Li, W. , Han, G. , Yang, Y. , Ma, Y. , Li, H. et al. (2013) Development of a seedling clone with high regeneration capacity and susceptibility to *Agrobacterium* in apple. Scientia Horticulturae, 164, 202–208.

[mpp13248-bib-0010] Dharmasiri, N. , Dharmasiri, S. , Weijers, D. , Lechner, E. , Yamada, M. , Hobbie, L. et al. (2005) Plant development is regulated by a family of auxin receptor F box proteins. Developmental Cell, 9, 109–119. 15992545 10.1016/j.devcel.2005.05.014

[mpp13248-bib-0011] Dreher, K. & Callis, J. (2007) Ubiquitin, hormones and biotic stress in plants. Annals of Botany, 99, 787–822. 17220175 10.1093/aob/mcl255 PMC2802907

[mpp13248-bib-0012] Du, Y. , Mpina, M.H. , Birch, P.R. , Bouwmeester, K. & Govers, F. (2015) *Phytophthora infestans* RXLR effector AVR1 interacts with exocyst component Sec5 to manipulate plant immunity. Plant Physiology, 169, 1975–1990. 26336092 10.1104/pp.15.01169 PMC4634092

[mpp13248-bib-0013] Fabian, M. , Gao, M. , Zhang, X.‐N. , Shi, J. , Kim, S.‐H. , Patel, P. et al. (2021) The flowering time regulator FLK controls pathogen defense in *Arabidopsis thaliana* . bioRxiv. 10.1101/2021.01.10.426133 [preprint]. PMC10069895 36662556

[mpp13248-bib-0014] Fujisaki, K. & Ishikawa, M. (2008) Identification of an *Arabidopsis thaliana* protein that binds to tomato mosaic virus genomic RNA and inhibits its multiplication. Virology, 380, 402–411. 18762309 10.1016/j.virol.2008.07.033

[mpp13248-bib-0015] Guan, Q. , Wen, C. , Zeng, H. & Zhu, J. (2013) A KH domain‐containing putative RNA‐binding protein is critical for heat stress‐responsive gene regulation and thermotolerance in *Arabidopsis* . Molecular Plant, 6, 386–395. 23087326 10.1093/mp/sss119

[mpp13248-bib-0016] Heath, M.C. (2000) Hypersensitive response‐related death. Plant Molecular Biology, 44, 321–334. 11199391 10.1023/a:1026592509060

[mpp13248-bib-0017] Hemetsberger, C. , Herrberger, C. , Zechmann, B. , Hillmer, M. & Doehlemann, G. (2012) The *Ustilago maydis* effector Pep1 suppresses plant immunity by inhibition of host peroxidase activity. PLoS Pathogens, 8, e1002684. 22589719 10.1371/journal.ppat.1002684 PMC3349748

[mpp13248-bib-0018] Hu, M. , Liu, Y. , Yu, K. & Liu, X. (2014) Decreasing the amount of trypsin in in‐gel digestion leads to diminished chemical noise and improved protein identifications. Journal of Proteomics, 109, 16–25. 24984109 10.1016/j.jprot.2014.06.017

[mpp13248-bib-0019] Jacobson, M.D. (1996) Reactive oxygen species and programmed cell death. Trends in Biochemical Sciences, 21, 83–86. 8882579

[mpp13248-bib-0020] Jing, M. , Guo, B. , Li, H. , Yang, B. , Wang, H. , Kong, G. et al. (2016) A *Phytophthora sojae* effector suppresses endoplasmic reticulum stress‐mediated immunity by stabilizing plant binding immunoglobulin proteins. Nature Communications, 7, 11685. 10.1038/ncomms11685 PMC4895818 27256489

[mpp13248-bib-0021] Jones, J.D. & Dangl, J.L. (2006) The plant immune system. Nature, 444, 323–329. 17108957 10.1038/nature05286

[mpp13248-bib-0022] Kanzaki, H. , Saitoh, H. , Takahashi, Y. , Berberich, T. , Ito, A. , Kamoun, S. et al. (2008) NbLRK1, a lectin‐like receptor kinase protein of *Nicotiana benthamiana*, interacts with *Phytophthora infestans* INF1 elicitin and mediates INF1‐induced cell death. Planta, 228, 977–987. 18682978 10.1007/s00425-008-0797-y

[mpp13248-bib-0023] Karlsson, P. , Christie, M.D. , Seymour, D.K. , Wang, H. , Wang, X. , Hagmann, J. et al. (2015) KH domain protein RCF3 is a tissue‐biased regulator of the plant miRNA biogenesis cofactor HYL1. Proceedings of the National Academy of Sciences of the United States of America, 112, 14096–14101. 26512101 10.1073/pnas.1512865112 PMC4653147

[mpp13248-bib-0024] Kumar, S. , Stecher, G. & Tamura, K. (2016) MEGA7: molecular evolutionary genetics analysis version 7.0 for bigger datasets. Molecular Biology and Evolution, 33, 1870–1874. 27004904 10.1093/molbev/msw054 PMC8210823

[mpp13248-bib-0025] Lamb, C. & Dixon, R.A. (1997) The oxidative burst in plant disease resistance. Annual Review of Plant Biology, 48, 251–275. 10.1146/annurev.arplant.48.1.251 15012264

[mpp13248-bib-0026] Lee, K. & Kang, H. (2016) Emerging roles of RNA‐binding proteins in plant growth, development, and stress responses. Molecules and Cells, 39, 179–185. 26831454 10.14348/molcells.2016.2359 PMC4794599

[mpp13248-bib-0027] Levine, A. , Tenhaken, R. , Dixon, R. & Lamb, C. (1994) H_2_O_2_ from the oxidative burst orchestrates the plant hypersensitive disease resistance response. Cell, 79, 583–593. 7954825 10.1016/0092-8674(94)90544-4

[mpp13248-bib-0028] Li, Z. , Yin, Z. , Fan, Y. , Xu, M. , Kang, Z. & Huang, L. (2015) Candidate effector proteins of the necrotrophic apple canker pathogen *Valsa mali* can suppress BAX‐induced PCD. Frontiers in Plant Science, 6, 579. 26284095 10.3389/fpls.2015.00579 PMC4515548

[mpp13248-bib-0029] Li, H. , Wang, H. , Jing, M. , Zhu, J. , Guo, B. , Wang, Y. et al. (2018) A *Phytophthora* effector recruits a host cytoplasmic transacetylase into nuclear speckles to enhance plant susceptibility. eLife, 7, e40039. 30346270 10.7554/eLife.40039 PMC6249003

[mpp13248-bib-0030] Lim, M.H. , Kim, J. , Kim, Y.S. , Chung, K.S. , Seo, Y.H. , Lee, I. et al. (2004) A new *Arabidopsis* gene, *FLK*, encodes an RNA binding protein with K homology motifs and regulates flowering time via FLOWERING LOCUS C. The Plant Cell, 16, 731–740. 14973162 10.1105/tpc.019331 PMC385284

[mpp13248-bib-0031] Lin, Y. , Hu, Q. , Zhou, J. , Yin, W. , Yao, D. , Shao, Y. et al. (2021) *Phytophthora sojae* effector Avr1d functions as an E2 competitor and inhibits ubiquitination activity of GmPUB13 to facilitate infection. Proceedings of the National Academy of Sciences of the United States of America, 118, e2018312118. 33658365 10.1073/pnas.2018312118 PMC7958378

[mpp13248-bib-0032] Lu, D. , He, P. & Shan, L. (2010) Bacterial effectors target BAK1‐associated receptor complexes: one stone two birds. Communicative & Integrative Biology, 3, 80–83. 20585495 10.4161/cib.3.2.10301 PMC2889959

[mpp13248-bib-0033] Ma, T. , Chen, S. , Liu, J. , Fu, P. , Wu, W. , Song, S. et al. (2021) *Plasmopara viticola* effector PvRXLR111 stabilizes VvWRKY40 to promote virulence. Molecular Plant Pathology, 22, 231–242. 33253483 10.1111/mpp.13020 PMC7814959

[mpp13248-bib-0034] Macho, A.P. & Zipfel, C. (2014) Plant PRRs and the activation of innate immune signaling. Molecular Cell, 54, 263–272. 24766890 10.1016/j.molcel.2014.03.028

[mpp13248-bib-0035] McLellan, H. , Chen, K. , He, Q. , Wu, X. , Boevink, P.C. , Tian, Z. et al. (2020) The ubiquitin E3 ligase PUB17 positively regulates immunity by targeting a negative regulator, KH17, for degradation. Plant Communications, 1, 100020. 32715295 10.1016/j.xplc.2020.100020 PMC7371183

[mpp13248-bib-0036] Murphy, F. , He, Q. , Armstrong, M. , Giuliani, L.M. , Boevink, P.C. , Zhang, W. et al. (2018) The potato MAP3K StVIK is required for the *Phytophthora infestans* RXLR effector Pi17316 to promote disease. Plant Physiology, 177, 398–410. 29588335 10.1104/pp.18.00028 PMC5933144

[mpp13248-bib-0037] Nayyar, H. & Chander, S. (2004) Protective effects of polyamines against oxidative stress induced by water and cold stress in chickpea. Journal of Agronomy and Crop Science, 190, 355–365.

[mpp13248-bib-0038] Nicastro, G. , Taylor, I.A. & Ramos, A. (2015) KH‐RNA interactions: back in the groove. Current Opinion in Structural Biology, 30, 63–70. 25625331 10.1016/j.sbi.2015.01.002

[mpp13248-bib-0039] Ortuno‐Miquel, S. , Rodriguez‐Cazorla, E. , Zavala‐Gonzalez, E.A. , Martinez‐Laborda, A. & Vera, A. (2019) *Arabidopsis* HUA ENHANCER 4 delays flowering by upregulating the MADS‐box repressor genes FLC and MAF4. Scientific Reports, 9, 1478. 30728422 10.1038/s41598-018-38327-3 PMC6365585

[mpp13248-bib-0040] Park, C.H. , Chen, S. , Shirsekar, G. , Zhou, B. , Khang, C.H. , Songkumarn, P. et al. (2012) The *Magnaporthe oryzae* effector AvrPiz‐t targets the RING E3 ubiquitin ligase APIP6 to suppress pathogen‐associated molecular pattern‐triggered immunity in rice. The Plant Cell, 24, 4748–4762. 23204406 10.1105/tpc.112.105429 PMC3531864

[mpp13248-bib-0041] Petrov, V.D. & Van Breusegem, F. (2012) Hydrogen peroxide—a central hub for information flow in plant cells. AoB PLANTS, 2012, pls014. 22708052 10.1093/aobpla/pls014 PMC3366437

[mpp13248-bib-0042] Pontier, D. , Godiard, L. , Marco, Y. & Roby, D. (1994) Hsr203j, a tobacco gene whose activation is rapid, highly localized and specific for incompatible plant/pathogen interactions. The Plant Journal, 5, 507–521. 8012404 10.1046/j.1365-313x.1994.5040507.x

[mpp13248-bib-0043] Pontier, D. , Tronchet, M. , Rogowsky, P. , Lam, E. & Roby, D. (1998) Activation of *hsr*203, a plant gene expressed during incompatible plant–pathogen interactions, is correlated with programmed cell death. Molecular Plant‐Microbe Interactions, 11, 544–554. 9612953 10.1094/MPMI.1998.11.6.544

[mpp13248-bib-0044] Pradhan, A. , Ghosh, S. , Sahoo, D. & Jha, G. (2021) Fungal effectors, the double edge sword of phytopathogens. Current Genetics, 67, 27–40. 33146780 10.1007/s00294-020-01118-3

[mpp13248-bib-0045] Qi, G. , Chen, H. , Wang, D. , Zheng, H. , Tang, X. , Guo, Z. et al. (2021) The BZR1‐EDS1 module regulates plant growth‐defense coordination. Molecular Plant, 14, 2072–2087. 34416351 10.1016/j.molp.2021.08.011

[mpp13248-bib-0046] Ripoll, J.J. , Ferrandiz, C. , Martinez‐Laborda, A. & Vera, A. (2006) PEPPER, a novel K‐homology domain gene, regulates vegetative and gynoecium development in *Arabidopsis* . Developmental Biology, 289, 346–359. 16356489 10.1016/j.ydbio.2005.10.037

[mpp13248-bib-0047] Ripoll, J.J. , Rodriguez‐Cazorla, E. , Gonzalez‐Reig, S. , Andujar, A. , Alonso‐Cantabrana, H. , Perez‐Amador, M.A. et al. (2009) Antagonistic interactions between *Arabidopsis* K‐homology domain genes uncover PEPPER as a positive regulator of the central floral repressor FLOWERING LOCUS C. Developmental Biology, 333, 251–262. 19576878 10.1016/j.ydbio.2009.06.035

[mpp13248-bib-0048] Rodriguez‐Cazorla, E. , Ripoll, J.J. , Andujar, A. , Bailey, L.J. , Martinez‐Laborda, A. , Yanofsky, M.F. et al. (2015) K‐homology nuclear ribonucleoproteins regulate floral organ identity and determinacy in arabidopsis. PLoS Genetics, 11, e1004983. 25658099 10.1371/journal.pgen.1004983 PMC4450054

[mpp13248-bib-0049] Rufian, J.S. , Rueda‐Blanco, J. , Lopez‐Marquez, D. , Macho, A.P. , Beuzon, C.R. & Ruiz‐Albert, J. (2021) The bacterial effector HopZ1a acetylates MKK7 to suppress plant immunity. New Phytologist, 231, 1138–1156. 33960430 10.1111/nph.17442

[mpp13248-bib-0050] Schafer, F.Q. & Buettner, G.R. (2001) Redox environment of the cell as viewed through the redox state of the glutathione disulfide/glutathione couple. Free Radical Biology and Medicine, 30, 1191–1212. 11368918 10.1016/s0891-5849(01)00480-4

[mpp13248-bib-0051] Shetty, N.P. , Jørgensen, H.J.L. , Jensen, J.D. , Collinge, D.B. & Shetty, H.S. (2008) Roles of reactive oxygen species in interactions between plants and pathogens. European Journal of Plant Pathology, 121, 267–280.

[mpp13248-bib-0052] Spoel, S.H. & Dong, X. (2012) How do plants achieve immunity? Defence without specialized immune cells. Nature Reviews Immunology, 12, 89–100. 10.1038/nri3141 22273771

[mpp13248-bib-0053] Takahashi, Y. , Berberich, T. , Yamashita, K. , Uehara, Y. , Miyazaki, A. & Kusano, T. (2004) Identification of tobacco *HIN1* and two closely related genes as spermine‐responsive genes and their differential expression during the *Tobacco mosaic virus*‐induced hypersensitive response and during leaf‐ and flower‐senescence. Plant Molecular Biology, 54, 613–622. 15316293 10.1023/B:PLAN.0000038276.95539.39

[mpp13248-bib-0054] Tanaka, S. , Brefort, T. , Neidig, N. , Djamei, A. , Kahnt, J. , Vermerris, W. et al. (2014) A secreted *Ustilago maydis* effector promotes virulence by targeting anthocyanin biosynthesis in maize. eLife, 3, e01355. 24473076 10.7554/eLife.01355 PMC3904489

[mpp13248-bib-0055] Thatcher, L.F. , Kamphuis, L.G. , Hane, J.K. , Onate‐Sanchez, L. & Singh, K.B. (2015) The *Arabidopsis* KH‐domain RNA‐binding protein ESR1 functions in components of jasmonate signalling, unlinking growth restraint and resistance to stress. PLoS One, 10, e0126978. 25985302 10.1371/journal.pone.0126978 PMC4436139

[mpp13248-bib-0056] Trachootham, D. , Alexandre, J. & Huang, P. (2009) Targeting cancer cells by ROS‐mediated mechanisms: a radical therapeutic approach? Nature Reviews Drug Discovery, 8, 579–591. 19478820 10.1038/nrd2803

[mpp13248-bib-0057] Vega‐Sanchez, M.E. , Zeng, L. , Chen, S. , Leung, H. & Wang, G.L. (2008) SPIN1, a K homology domain protein negatively regulated and ubiquitinated by the E3 ubiquitin ligase SPL11, is involved in flowering time control in rice. The Plant Cell, 20, 1456–1469. 18586868 10.1105/tpc.108.058610 PMC2483366

[mpp13248-bib-0058] Wang, Y.Q. , Lin, A.H. , Loake, G.J. & Chu, C.C. (2013) H_2_O_2_‐induced leaf cell death and the crosstalk of reactive nitric/oxygen species. Journal of Integrative Plant Biology, 55, 202–208. 23331502 10.1111/jipb.12032

[mpp13248-bib-0059] Wang, W. , Nie, J. , Lv, L. , Gong, W. , Wang, S. , Yang, M. et al. (2021) A *Valsa mali* effector protein 1 targets apple (*Malus domestica*) pathogenesis‐related 10 protein to promote virulence. Frontiers in Plant Science, 12, 741342. 34691119 10.3389/fpls.2021.741342 PMC8528966

[mpp13248-bib-0060] Wojtaszek, P. (1997) Oxidative burst: an early plant response to pathogen infection. The Biochemical Journal, 322, 681–692. 9148737 10.1042/bj3220681 PMC1218243

[mpp13248-bib-0061] Wu, G. , Shortt, B.J. , Lawrence, E.B. , Levine, E.B. , Fitzsimmons, K.C. & Shah, D.M. (1995) Disease resistance conferred by expression of a gene encoding H_2_O_2_‐generating glucose oxidase in transgenic potato plants. The Plant Cell, 7, 1357–1368. 8589621 10.1105/tpc.7.9.1357 PMC160957

[mpp13248-bib-0062] Xiao, S. , Brown, S. , Patrick, E. , Brearley, C. & Turner, J.G. (2003) Enhanced transcription of the *Arabidopsis* disease resistance genes *RPW8.1* and *RPW8.2* via a salicylic acid‐dependent amplification circuit is required for hypersensitive cell death. The Plant Cell, 15, 33–45. 12509520 10.1105/tpc.006940 PMC143449

[mpp13248-bib-0063] Yan, Z. , Jia, J. , Yan, X. , Shi, H. & Han, Y. (2017) *Arabidopsis* KHZ1 and KHZ2, two novel non‐tandem CCCH zinc‐finger and K‐homolog domain proteins, have redundant roles in the regulation of flowering and senescence. Plant Molecular Biology, 95, 549–565. 29076025 10.1007/s11103-017-0667-8

[mpp13248-bib-0064] Yang, Q. , Huai, B. , Lu, Y. , Cai, K. , Guo, J. , Zhu, X. et al. (2020) A stripe rust effector Pst18363 targets and stabilises TaNUDX23 that promotes stripe rust disease. New Phytologist, 225, 880–895. 31529497 10.1111/nph.16199

[mpp13248-bib-0065] Yin, Z. (2018) Analysis of whole genome and mechanisms of the recongnition of two secreted proteins of pathogenic fungus *Valsa mali* by plants. [PhD thesis]. Northwest Agriculture and Forest University.

[mpp13248-bib-0066] Zhang, M. , Li, Q. , Liu, T. , Liu, L. , Shen, D. , Zhu, Y. et al. (2015) Two cytoplasmic effectors of *Phytophthora sojae* regulate plant cell death via interactions with plant catalases. Plant Physiology, 167, 164–175. 25424308 10.1104/pp.114.252437 PMC4281015

[mpp13248-bib-0067] Zipfel, C. & Oldroyd, G.E. (2017) Plant signalling in symbiosis and immunity. Nature, 543, 328–336. 28300100 10.1038/nature22009

