# Supplementary figures and images for: Valsa mali secretes an effector protein VmEP1 to target a K homology domain‐containing protein for virulence in apple
Source: Mol Plant Pathol. 2022 Jul 18;23(11):1577–91. doi: 10.1111/mpp.13248 (PMC9562843; doi:10.1111/mpp.13248)

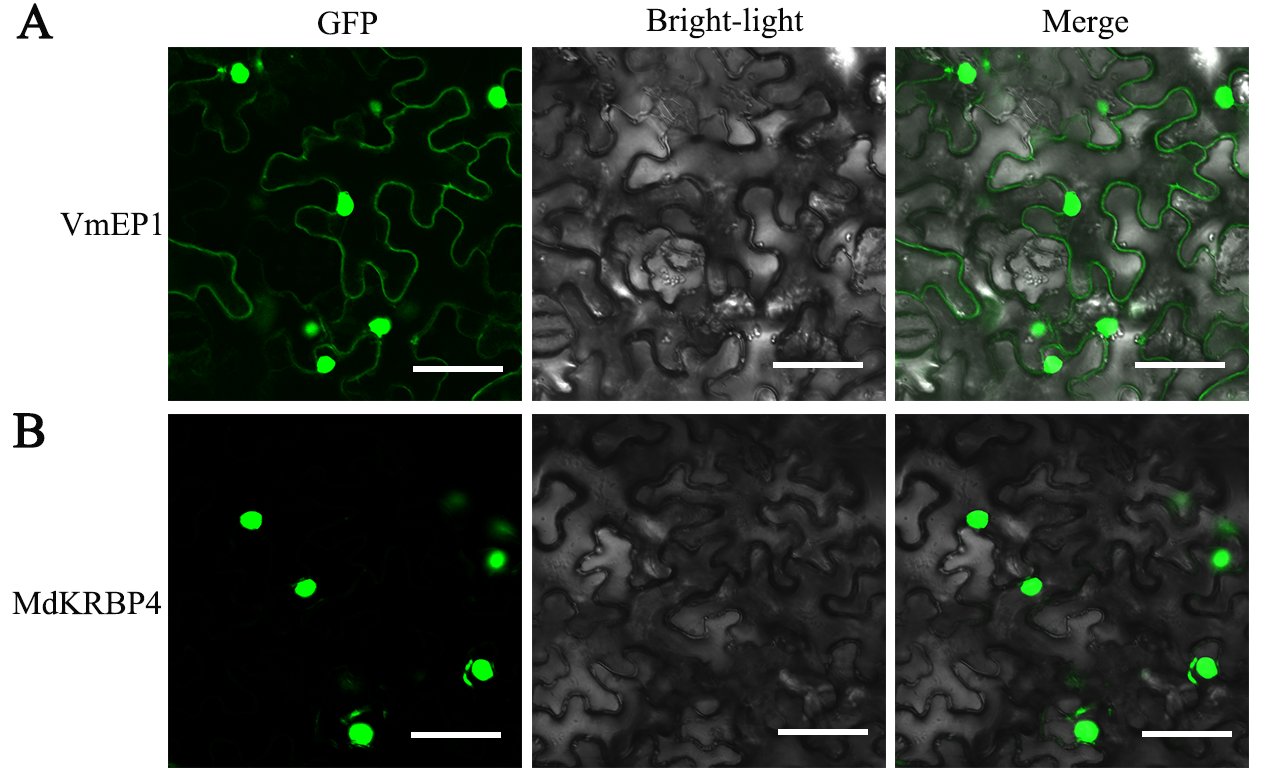

Supplement: Supplementary file 1 — FIGURE S1 Expression of VmEP1 and MdKRBP4 in plants. (a) Subcellular localization of VmEP1 was visualized by confocal microscopy in Nicotiana benthamiana. (b) Subcellular localization of MdKRBP4 was visualized by confocal microscopy in N. benthamiana. Scale bar represents 40 μm [file MPP-23-1577-s004.tif]

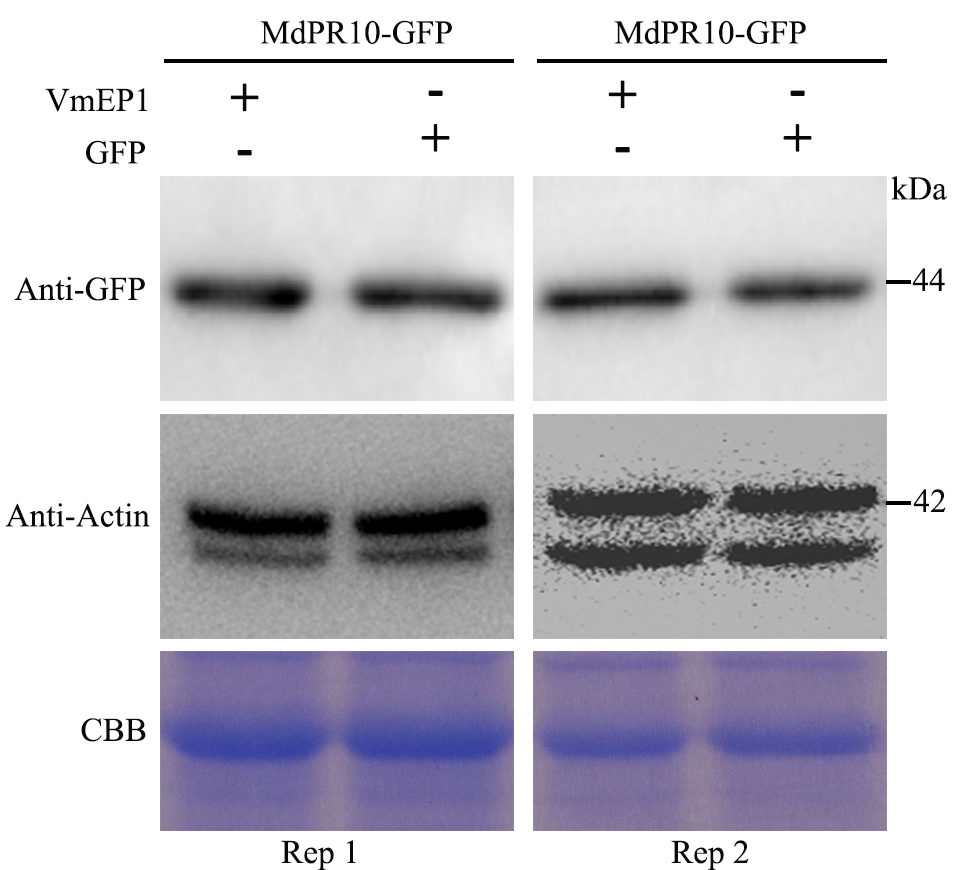

Supplement: Supplementary file 2 — FIGURE S2 The effect of VmEP1 on MdPR10 protein accumulation was analysed by western blot. Total protein was extracted from Nicotiana benthamiana leaves expressing MdPR10‐GFP/VmEP1‐HA or MdPR10‐GFP/GFP and immunoblotted using anti‐GFP to detect MdPR10. Anti‐actin and Coomassie brilliant blue (CBB) staining were used as loading control [file MPP-23-1577-s005.tif]

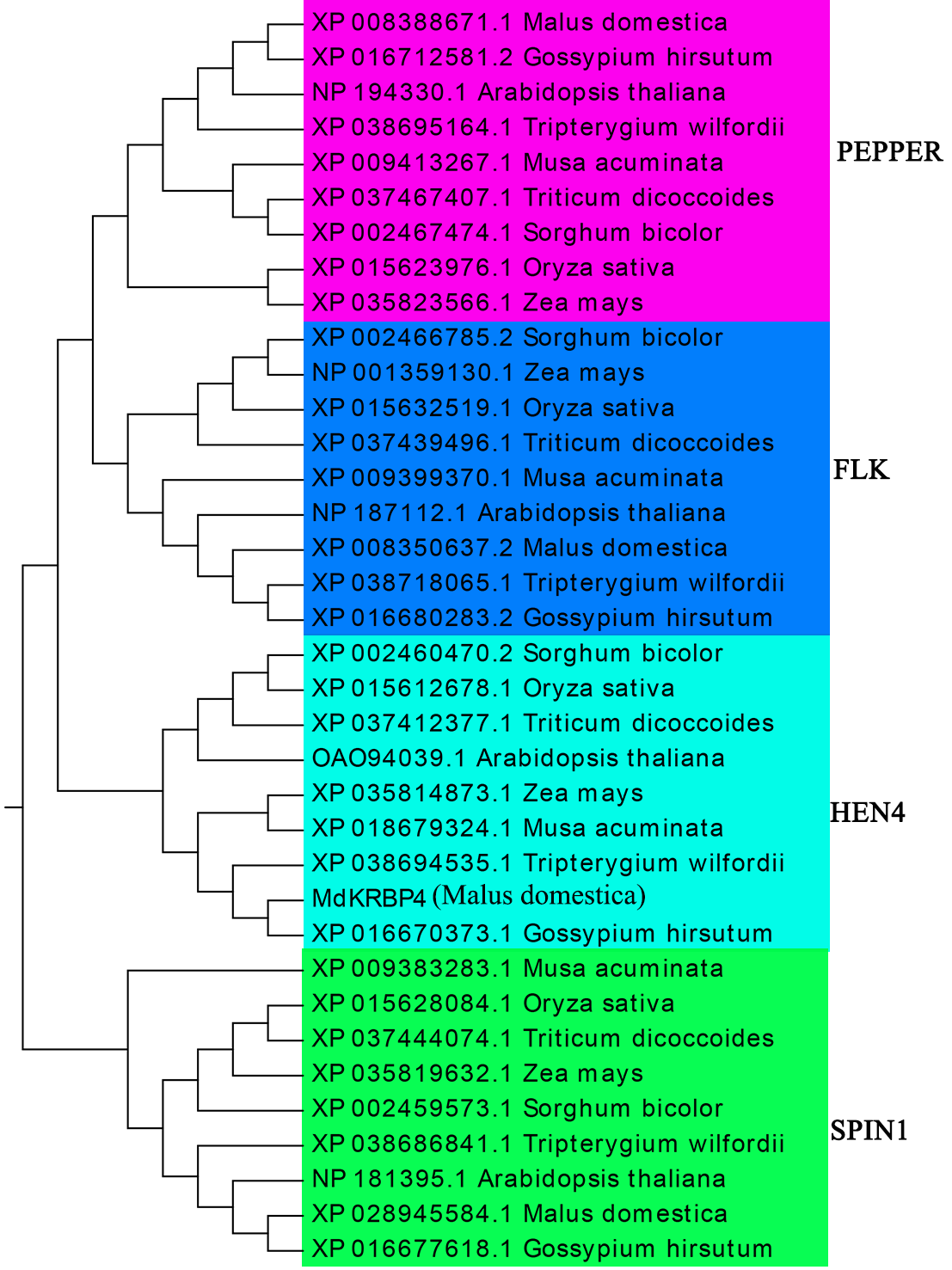

Supplement: Supplementary file 3 — FIGURE S3 Phylogenetic tree constructed using amino acid sequences of KH domain‐containing proteins. Shown is the phylogeny of MdKRBP4 and its homologous sequences from selected species including Cruciferae (Arabidopsis thaliana), Gramineae (Triticum dicoccoides, Zea mays, Oryza sativa, Sorghum bicolor), Musaceae (Musa acuminata), Celastraceae (Tripterygium wilfordii), Rosaceae (Malus domestica), and Malvaceae (Gossypium hirsutum). The tree was constructed with the neighbour‐joining method [file MPP-23-1577-s006.tif]

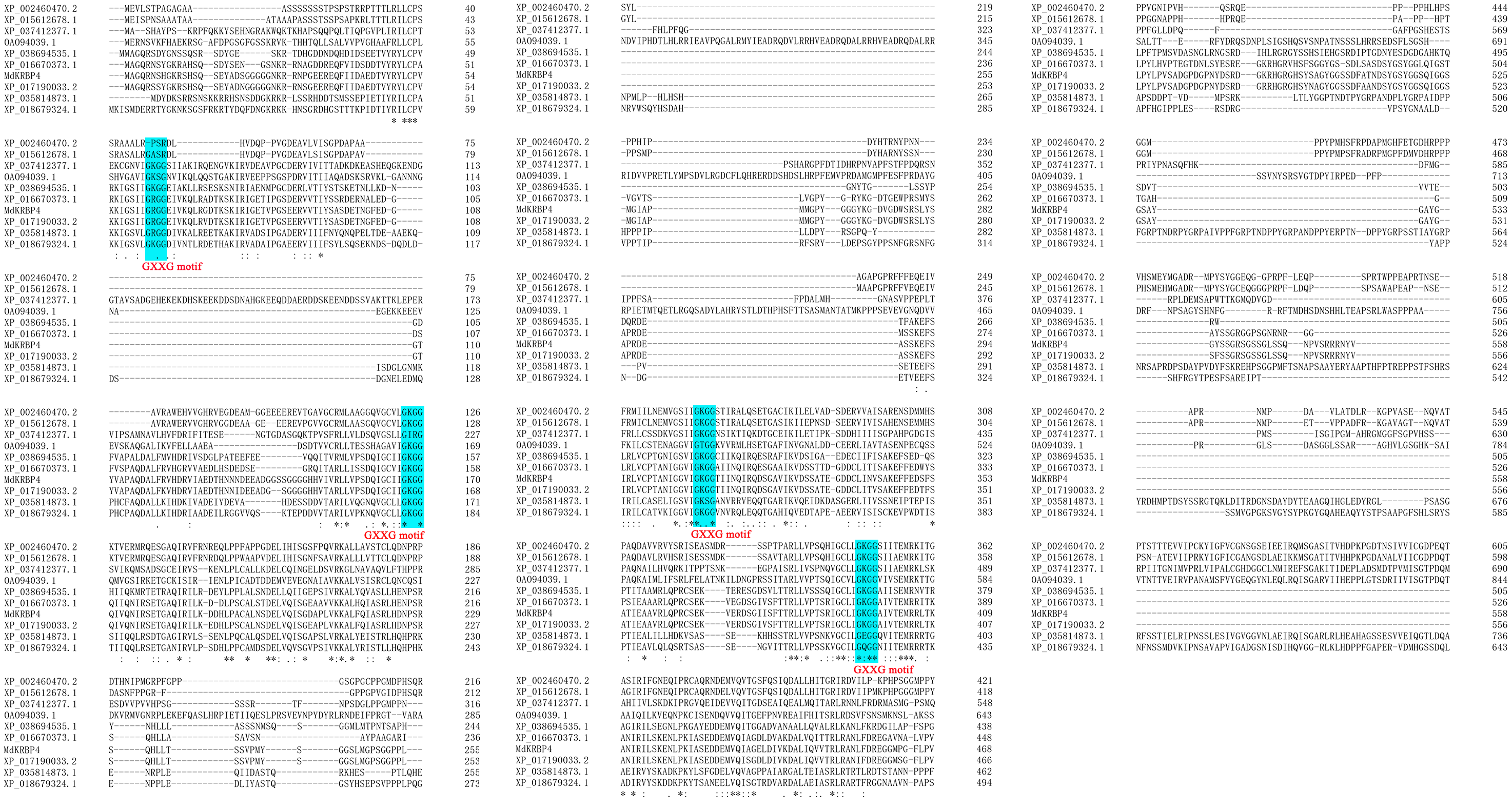

Supplement: Supplementary file 4 — FIGURE S4 Multiple alignment of HEN4 sequences. The amino acid sequences were aligned by Clustal Omega, and conserved GXXG motifs are highlighted in different colours [file MPP-23-1577-s003.tif]
