# Supplementary material for: Valsa mali secretes an effector protein VmEP1 to target a K homology domain‐containing protein for virulence in apple
Source: Mol Plant Pathol. 2022 Jul 18;23(11):1577–91. doi: 10.1111/mpp.13248 (PMC9562843; doi:10.1111/mpp.13248)
Supplement: Supplementary file 5 — TABLE S1 NCBI BLASTp results of AtHEN4 in Malus domestica (taxid:3750) [file MPP-23-1577-s002.docx]

**Supplementary Table 1**. VmEP1-associating proteins detected by H2Y

| ACCESSION | Discription |
| --- | --- |
| AAX18320.1 | *Malus domestica* major allergen Mal d 1.03E |
| XM_029102041 | *Malus domestica* SWI/SNF complex subunit SWI3D-like |
| XR_003769028 | *Malus domestica* developmentally-regulated G-protein 3 |
| XR_003769179 | *Malus domestica* methylenetetrahydrofolate reductase 2-like |
| XM_029089771 | *Malus domestica* glutamate-tRNA ligase, cytoplasmic-like |
| XM_008344245 | *Malus domestica* VQ motif-containing protein 22 |
| XR_003774273 | *Malus domestica* acidic endochitinase SE2-like |
| XM_008367569 | *Malus domestica* NADPH-dependent aldehyde reductase-like protein |
| XM_008345684 | *Malus domestica* trafficking protein particle complex subunit 5 |
| XM_008372720 | *Malus domestica* auxin-responsive protein IAA30-like |
| XP_008390852 | PREDICTED: putative methyltransferase C9orf114 |
| **XM_029110241** | ***Malus domestica* KH domain-containing protein At4g18375** |
| XM_008376128 | PREDICTED: *Malus domestica* BTB/POZ and TAZ domain-containing protein 1 |
| XP_008369178 | *Malus domestica* uncharacterized protein LOC103432754 |
| XM_029105357 | *Malus domestica* uncharacterized LOC103438788 |
| XR_003768977 | *Malus domestica* uncharacterized LOC103401657 |
