# Supplementary material for: Valsa mali secretes an effector protein VmEP1 to target a K homology domain‐containing protein for virulence in apple
Source: Mol Plant Pathol. 2022 Jul 18;23(11):1577–91. doi: 10.1111/mpp.13248 (PMC9562843; doi:10.1111/mpp.13248)
Supplement: Supplementary file 6 — TABLE S2 Primers used in this study [file MPP-23-1577-s001.docx]

| **Description** | **Query Cover** | **E value** | **Per. Ident** | **Accession** |
| --- | --- | --- | --- | --- |
| KH domain-containing protein HEN4 isoform X2 [Malus domestica] | 75% | 1e-72 | 41.33% | XP_008358399.2 |
| KH domain-containing protein HEN4 isoform X1 [Malus domestica] | 75% | 4e-71 | 41.23% | XP_008358398.2 |
| LOW QUALITY PROTEIN: KH domain-containing protein HEN4 [Malus domestica] | 73% | 2e-70 | 41.45% | XP_028965343.1 |
| KH domain-containing protein HEN4-like isoform X1 [Malus domestica] | 69% | 8e-53 | 32.95% | XP_028964035.1 |
| KH domain-containing protein HEN4-like isoform X2 [Malus domestica] | 69% | 2e-51 | 31.66% | XP_028964036.1 |
| KH domain-containing protein HEN4-like [Malus domestica] | 46% | 7e-37 | 40.66% | XP_008378683.2 |
| KH domain-containing protein HEN4 [Malus domestica] | 42% | 2e-30 | 37.02% | XP_017190033.2 |
| KH domain-containing protein HEN4-like [Malus domestica] | 42% | 3e-29 | 39.08% | XP_028954217.1 |

**Supplementary Table 2**. Results of AtHEN4 with NCBI blastp in *Malus domestica* (taxid:3750).
