# Supplementary material for: Valsa mali secretes an effector protein VmEP1 to target a K homology domain‐containing protein for virulence in apple
Source: Mol Plant Pathol. 2022 Jul 18;23(11):1577–91. doi: 10.1111/mpp.13248 (PMC9562843; doi:10.1111/mpp.13248)
Supplement: Supplementary file 7 — TABLE S3 PCR primers used in this study [file MPP-23-1577-s007.docx]

**Supplementary Table 3**. Polymerase chain reaction primers used in this study.

| **Primer name** | **Sequence** | **Purpose** |
| --- | --- | --- |
| PK7-MdKRBP4-F | GGGGACAAGTTTGTACAAAAAAGCAGGCTTC CATTCTCAGCAACTATTGAAGC | Clone MdKRBP4 to PK2 for expression in *M. domestica* |
| PK7-MdKRBP4-F | GGGGACCACTTTGTACAAGAAAGCTGGGTC CCTTAACAATATCAAGTTCTC |  |
| PCH-VmEP1-F | TTACAATTATCGATACAATG TACCCATACGACGTCCCAGACTACGCT ATGAGCCTTGTTACTACCTGCG | Clone VmEP1 to PCH86988 for expression in *N. benthamiana* |
| PCH-VmEP1-R | CTCATTAAAGCAGGACAAGC TCAGTCTACCGAACATGTCTGTGG |  |
| nYFP–MdKRBP4-F | GTGCCGCGCGGCAGCCATATG ATGGCAGGTCAGAGGAATAGTC | Clone MdKRBP4 to nYFP for expression in *N. benthamiana* |
| nYFP–MdKRBP4-R | ACGGAGCTCGAATTCGGATCC AACATAGTTTCTCCTCCGAG |  |
| cYFP-VmEP1-F | GCCCAAGCTTCGACtctaga ATGAGCCTTGTTACTACCTGCG | Clone VmEP1 to cYFP for expression in *N. benthamiana* |
| cYFP-VmEP1-R | ACGCTGCCGTCCATggatcc TCAGTCTACCGAACATGTCTGTGG |  |
| 1302–MdKRBP4-F | GTGCCGCGCGGCAGCCATATG ATGGCAGGTCAGAGGAATAGTC | Clone MdKRBP4 to pCAMBIA1302 for expression in *N. benthamiana* |
| 1302–MdKRBP4-R | ACGGAGCTCGSAATTCGGATCC AACATAGTTTCTCCTCCGAG |  |
| qMdCAT2-F | GAACCCTAAGTCCCACATCC | qRT-PCR analysis in *M. domestica* |
| qMdCAT2-F | GAACTCCCAGGTCATCCAAT |  |
| qMdRBOHD-F | TTGCTGTTTATCCTGGAAATG |  |
| qMdRBOHD-F | GAGGTAATGGAGAACGGGTG |  |
| qMdHSR203J-F | AGCGGAGTAAGTCGCAGTTGG |  |
| qMdHSR203J-R | TTATGGGATGGTCCTTGGTGG |  |
| qMdHIN1-F | GGTGTACCAAGGCCACAAGGA |  |
| qMdHIN1-R | TGATGAGCATAAGAACCGACCC |  |
| qMdPR1-F | CGTGGGATGACAATGTAGCAGG |  |
| qMdPR1-R | GCAAGGTTTTCACCGTATGGC |  |
| qMdPR2-F | CCTGCCATCCAAAACATCCAC |  |
| qMdPR2-R | GCTGAAGTAAGGGTACACATTCACAA |  |
| qMdPR5-F | TGCGGCAAACGGGGGTC |  |
| qMdPR5-R | GCAGGGCAAGACGAGGGCT |  |
| qRT-NbHIN1-F | CCAACTTGAACGGAGCCTATTA | qRT-PCR analysis in *N. benthamiana* |
| qRT-NbHIN1-R | AGGCATCCAAAGAGACAACTAC |  |
| qRT-NbHSR203J-F | ACGCAGATTTCAACCGAGTAT |  |
| qRT-NbHSR203J-R | GCCAGTCGCATTGGAGATAA |  |
